# Schwann cell-derived extracellular vesicles promote periodontal tissue regeneration

**DOI:** 10.1186/s12951-026-04848-7

**Published:** 2026-07-26

**Authors:** Ting Zhang, Jiaying Liu, Xiyue Wang, Yu Wu, Mengjia Wang, Shuyu Cheng, Rong Liu, Shuang Zhang, Yangheng Zhang, Yin Xiao, Yanan Zhu, Fuhua Yan

**Affiliations:** 1 Nanjing Stomatological Hospital, Affiliated Hospital of Medical School, Institute of Stomatology, Nanjing University, Nanjing, China; 2 School of Medicine and Dentistry, Griffith University, QLD 4222 Gold Coast, Australia; 3 Department of Periodontology, Guiyang Hospital of Stomatology, Guiyang, 550002 GuiZhou China; 4 Institute for Biomedicine and Glycomics, Griffith University, QLD, 4222, Gold Coast, Australia; 5 Australian Centre for Precision Health and Technology (PRECISE), Griffith University, QLD, 4222, Gold Coast, Australia

**Keywords:** Schwann cells, Extracellular vesicles, Periodontal ligament stem cells, Osteogenic differentiation, Autophagy

## Abstract

Periodontitis is a common chronic inflammatory disease worldwide, and conventional therapies control inflammation but fail to achieve predictable regeneration. The inferior alveolar nerve (IAN) is a key peripheral nerve that governs mandibular bone homeostasis, and its disruption precipitates bone metabolic imbalance and impaired repair. However, the dynamic behavior of Schwann cells (SCs), the principal glial cells of this neural axis, and their mechanistic influence on periodontal ligament stem cells (PDLSCs) remain elusive. We established a rat IAN transection model and found that denervation induced progressive alveolar bone deterioration and compromised the osteogenic capacity of resident PDLSCs. Correlation analyses revealed that this bone deterioration coincided with progressive SC depletion in periodontal tissue, suggesting a link between Schwann cell loss and PDLSC dysfunction. Transcriptomic profiling of denervated PDLSCs revealed that dysregulated AMPK/mTOR signaling and suppressed autophagy are mechanisms underlying osteogenic impairment. Co-culture with SCs restored PDLSC proliferation and osteogenic differentiation, with SC-derived extracellular vesicles (SC-EVs) as important paracrine mediators of this effect. Mechanistically, SC-EVs delivered rno-miR-210-5p, which targeted the *DAP1* gene and suppressed its expression, thereby activating the AMPK/mTOR-autophagy axis and rescuing osteogenic potential. In a rat denervated mandibular defect model, SC-EVs significantly enhanced bone regeneration. Collectively, these findings suggest that Schwann cells and their EVs participate in the neural regulation of periodontal bone metabolism and may play an important role in periodontal regeneration.

**Supplementary Information:**

The online version contains supplementary material available at https://doi.org/10.1186/s12951-026-04848-7.

## Introduction

Periodontitis is a chronic infectious disease of periodontal supporting tissues initiated by dental plaque biofilms. As a prevalent oral condition, it poses a significant global public health challenge. Epidemiological studies indicate that approximately 62% of adults are affected by periodontitis, and 23.6% of these cases are severe [1, 2]. Alveolar bone loss is the leading cause of tooth loss in adults [3]. Conventional clinical treatments, such as mechanical debridement and guided tissue regeneration, effectively control inflammation. However, predictable regeneration of the periodontal ligament-alveolar bone-cementum complex remains difficult to achieve with these therapies, underscoring their limitations [4]. From a developmental biology perspective, the formation of periodontal tissue depends on interactions between nerves and hard tissues. Consequently, strategies focusing solely on bone augmentation have inherent limitations. Therefore, reconstructing the nerve-bone regulatory network and restoring microenvironmental homeostasis are considered crucial for achieving true periodontal regeneration. In addition to their well-established roles in supporting nerve conduction and axonal regeneration [5, 6], Schwann cells (SCs) have recently been shown to play extensive roles in tissue repair [7]. SCs secrete multiple bioactive factors, including neurotrophic factors such as nerve growth factor (NGF) [8] and brain-derived neurotrophic factor (BDNF) [9], as well as extracellular vesicles (EVs) [10, 11], all of which have been implicated in bone metabolism and regeneration. Accumulating evidence has demonstrated that SCs promote digit regeneration [12], skin repair [13], maintenance of hematopoietic stem cell homeostasis [14], and enhance osteogenesis in mandibular bone repair [15], thus establishing their pivotal role as bridging cells in nerve-tissue regeneration.

Extracellular vesicles derived from Schwann cells (SC-EVs) are crucial functional mediators of glial cell paracrine signaling. They serve as key players in intercellular communication by transporting various bioactive cargos, including proteins, mRNAs, and miRNAs [16]. Emerging evidence indicates that SC-EVs can modulate bone homeostasis and stem cell differentiation, thus influencing tissue regeneration. For instance, SC-EVs have been shown to increase the osteogenic differentiation potential of bone marrow mesenchymal stem cells (BMSCs) and stimulate angiogenesis via the let-7c-5p/TGF-β signaling pathway [17]. Furthermore, SC-EV-derived miR-15b-5p has been reported to ameliorate impaired bone regeneration under diabetic conditions by modulating the Txnip signaling axis [18]. Moreover, the combination of SC-EVs with biomaterials (such as porous titanium alloys and biomimetic periosteum) significantly enhances bone repair efficacy [19]. MicroRNAs (miRNAs) serve as key post-transcriptional regulators of gene expression and constitute crucial cargo in EVs, through which they mediate tissue communication via paracrine and endocrine routes. Secreted miRNAs, particularly those encapsulated within EVs, facilitate paracrine and endocrine communication between tissues, thus regulating gene expression and function in distal recipient cells. Dysregulation of this process can contribute to tissue dysfunction, aging, and related pathologies [20]. Therefore, identifying specific miRNAs carried by EVs and their potential functional targets may help clarify the molecular basis of cross-tissue signaling and offer additional insight into disease pathogenesis.

However, the specific regulatory role of SC-EVs in periodontal regeneration remains unclear, particularly regarding the mechanisms underlying nerve-periodontal interactions within this highly innervated microenvironment. Accordingly, this study aimed to systematically investigate the dynamic changes in Schwann cells following inferior alveolar nerve (IAN) transection and their association with periodontal tissue remodeling. Furthermore, we sought to clarify the molecular mechanisms through which the denervated microenvironment impairs osteogenic differentiation of PDLSCs. Additionally, whether SC-EVs promote periodontal regeneration by regulating PDLSCs via specific signaling pathways was explored. These findings may provide experimental support for further exploration of nerve-derived EV-based approaches in periodontal regeneration research.

## Materials and methods

### Establishment of the rat inferior alveolar nerve transection model

These animal model procedures were approved by the Animal Ethics Committee of Nanjing Agricultural University (Approval No. PZW2022054) and conducted in strict compliance with institutional guidelines for animal welfare. Seventy-two 5-week-old male Sprague-Dawley rats (purchased from Charles River Laboratories) were acclimated for 1 week and randomly assigned to sham or denervation groups (*n* = 6 per group at each time point). Under isoflurane anesthesia, a 1.5 cm skin incision was made over the right mandibular angle. Following disinfection, the overlying muscles were bluntly dissected to expose the underlying bone surface, allowing visualization of the mandibular foramen and inferior alveolar nerve. In the denervation group, a 5‑mm segment of the IAN was resected to prevent axonal regeneration. The sham-operated group underwent surgical exposure of the IAN without transection. Surgical sites were copiously irrigated with saline and closed in layers with sutures. Postoperative antibiotics were administered subcutaneously for 3 days. Mandibular tissues were collected on postoperative days 1, 5, 7, 14, 21, and 28.

### Construction of the IAN transection and periodontal defect model in rats

The experimental procedure of this animal model was approved by the Animal Ethics Committee of Nanjing Agricultural University (PZW2022003). Eighteen 5-week-old male Sprague-Dawley rats were acclimated for 1 week. The animals were randomly divided into three groups (*n* = 6 per group): untreated control (Con), GelMA hydrogel alone (GelMA), and GelMA hydrogel loaded with SC-EVs (EVs+GelMA). The GelMA prepolymer solution was prepared using EFL-GM-30 and 0.5% (w/v) LAP (EFL, China). SC-EVs were isolated from the supernatant of RSC96 cells by ultracentrifugation, incorporated into the GelMA solution, and gently mixed to obtain a final EV concentration of 1 µg/µl. Under isoflurane anesthesia, unilateral IAN transection was performed as described above. A standardized periodontal fenestration defect was subsequently created on the buccal surface of the ipsilateral mandible, measuring 2 mm in apicocoronal height, 5 mm in mesiodistal width (extending parallel to the alveolar crest and 0.5 mm apical to it), and 0.8**–**1 mm in depth (exposing the cementoenamel junction and partially removing the cementum layer). The defect was thoroughly irrigated with saline to remove debris and subsequently filled with a blood clot. According to the group assignment, the corresponding hydrogel was placed into the defect and photopolymerized for 30 s using a portable light-curing unit positioned 10 cm away. The surgical wound was closed with layered sutures, and postoperative antibiotics were administered for 3 days. Mandibular tissues were harvested 21 days after surgery, following euthanasia of the rats.

### Micro-computed tomography (micro-CT) analysis

Mandibular samples were fixed in 4% paraformaldehyde for 24 h and scanned with a high-resolution micro-CT system (Bruker, Belgium) at an isotropic voxel size of 18 μm. Three-dimensional (3D) reconstruction and morphometric analysis were conducted using CTAn software (Bruker, Belgium). The evaluated parameters included bone volume fraction (BV/TV), bone mineral density (BMD), trabecular thickness (Tb.Th), trabecular separation (Tb. Sp), and trabecular number (Tb. N). Investigators performing micro-CT reconstruction and quantitative analyses were blinded to group allocation, and identical scanning settings, thresholding criteria, and VOI definitions were applied to all samples within the same experiment.

### Histological and immunohistochemical (IHC) analysis

Decalcified rat mandibles were embedded in paraffin and serially sectioned into 4–5 μm thick slices. Sections were stained with hematoxylin and eosin (H&E) or Masson’s trichrome according to standard protocols. For IHC, deparaffinized sections were subjected to antigen retrieval using urea buffer, followed by enzymatic digestion with trypsin and pepsin. After blocking with normal serum, sections were incubated overnight at 4 °C with the following primary antibodies: anti-S100 (Abcam, ab4066; 1:100), anti-Runx2 (Proteintech, 20700-1-AP; 1:200), and anti-LC3 (Proteintech, 14600-1-AP; 1:200). Immunoreactivity was detected using an HRP-Polymer anti-Mouse/Rabbit IHC Kit (MXB, China) and visualized with 3, 3’-diaminobenzidine (DAB) substrate (MXB, China). All the stained sections were digitized using a Pannoramic 250 slide scanner (3D HISTECH Ltd., Budapest, Hungary). Histological quantification was performed on the digitized images using standardized criteria. The investigators responsible for image analysis were blinded to group allocation during data collection and preliminary quantification.

### Cell isolation and culture

Rat Schwann cells were obtained from the RSC96 cell line, a spontaneously transformed rat Schwann cell line from Cellcook Biotech Co., Ltd (Guangzhou, China). RSC96 cells were cultured in high-glucose Dulbecco’s modified Eagle’s medium (DMEM), supplemented with 10% fetal bovine serum (FBS).

Rat periodontal ligament stem cells (rPDLSCs) were isolated by enzymatic digestion. The rats were euthanized under anesthesia induced by an intraperitoneal injection of pentobarbital sodium (Merck Millipore, USA). Following extraction of the first and second molars, the mandibles were surface-disinfected in 75% ethanol, transferred to a biosafety cabinet, and rinsed with DMEM containing 5% penicillin-streptomycin. The periodontal ligament tissues from the middle third of the roots were carefully scraped and then digested in a solution containing collagenase type I (3 mg/mL) and neutral protease (4 mg/mL) at 37 °C for 1 h. The enzymatic digestion was quenched by adding an equal volume of complete culture medium. The cell suspension was filtered through a 70‑µm cell strainer and centrifuged at 1000 × g for 5 min, after which the pellet was resuspended in low-glucose DMEM supplemented with 10% FBS for culture. Cells between passages 2 and 4 were used for all subsequent experiments.

### Flow cytometry

PDLSCs (passages 2–4) were harvested by trypsinization, washed, and resuspended in phosphate-buffered saline (PBS) at a density of 1 × 10⁶ cells/mL. The cell aliquots were distributed into flow cytometry tubes and pelleted by centrifugation. The cell pellets were resuspended and incubated for 30 min at 4 °C in the dark with the following fluorochrome-conjugated anti-rat antibodies and their respective isotype controls (all from BioLegend, USA): FITC-conjugated anti-CD11b/c, APC-conjugated anti-CD45, FITC-conjugated anti-CD29, PerCP/Cyanine5.5-conjugated anti-CD90, APC-conjugated anti-CD105, and APC-conjugated anti-STRO-1. Following three washes with PBS, the cells were resuspended in PBS for immediate analysis on a BD FACS instrument (BD Biosciences, USA).

### Colony-forming unit (CFU) and cell proliferation assays

PDLSCs (passages 2**–**4) were digested into single-cell suspensions. For colony formation, 1,000 cells were seeded per 10-cm dish and cultured for 2**–**3 weeks. Colonies (> 50 cells) were fixed with 4% paraformaldehyde, stained with 0.1% crystal violet, and counted to calculate the formation rates. Cell proliferation was assessed using a CCK-8 assay (Dojindo, Japan). To compare the proliferation of normal and denervated PDLSCs, cells were seeded into 96-well plates at 5, 000 cells/well and cultured for 1, 3, 5, 7, and 9 days. To evaluate the effect of SC-EVs on PDLSC proliferation, cells seeded at the same density were treated with SC-EVs at 0, 10, 30, 50, 100, or 200 µg/mL for 24 h. At the indicated time points, 10 µL CCK-8 reagent was added to each well containing 100 µL serum‑free medium (final volume 110 µL), and after 2 h of incubation, absorbance at 450 nm was measured, with blank wells used for background normalization.

### Osteogenic and adipogenic differentiation of PDLSCs

PDLSCs at passages 2**–**4 (P2**–**4) were seeded in 6-well plates. After the cells reached ~ 80% confluence, the culture medium was replaced with osteogenic differentiation medium or adipogenic differentiation medium. The osteogenic medium consisted of basal medium supplemented with 0.1 µM dexamethasone, 10 mM β-glycerophosphate, and 50 µg/mL L‑ascorbic acid (all from Sigma, USA). The adipogenic medium consisted of basal medium supplemented with 1 µM dexamethasone, 0.5 mM 3-isobutyl-1-methylxanthine (IBMX), 200 µM indomethacin, and 10 µg/mL insulin (Cyagen, China). The induction media were changed every 3 days.

### Alkaline phosphatase (ALP) staining and activity assay

After 7 days of osteogenic induction, rat PDLSCs were fixed with 4% neutral formaldehyde for 30 min and stained with BCIP/NBT (Beyotime, China) in the dark for 30 min to assess ALP activity. To measure ALP activity, the cells were lysed with 1% Triton X-100 (Beyotime, China) after 7 days of osteogenic induction. The cell lysates were incubated with p-nitrophenyl phosphate (pNPP) substrate solution at 37 °C for 5**–**10 min. The reaction was terminated by adding stop solution, and the absorbance was measured at 405 nm using a microplate reader. ALP activity, expressed as nmol of p-nitrophenol produced per min per mg of protein, was calculated using a p-nitrophenol standard curve and normalized to total protein concentration.

### Alizarin red S (ARS) staining and quantification

PDLSCs were fixed with 4% paraformaldehyde for 30 min after osteogenic induction and stained with Alizarin Red S (Cyagen, China) for 30 min at room temperature in the dark. After thorough washing, mineralized nodules were observed. The stained samples were eluted with 10% cetylpyridinium chloride (CPC) (Sigma, USA) under gentle agitation for 15–60 min. The resulting destaining solution was diluted 20-fold with 10% CPC, and its absorbance was measured at 562 nm. The calcium deposition level was quantified by comparison to a standard curve and normalized to the total protein content.

### Oil red O (ORO) staining

After adipogenic induction, the cells were washed three times with PBS and fixed with 4% neutral buffered formalin for 30 min at room temperature. Following fixation and subsequent rinses with PBS, the cells were incubated with 1 mL of Oil Red O working solution (Cyagen, China) for 30 min at room temperature. The cells were subsequently washed again with PBS and examined under a microscope to observe lipid droplet formation.

### Quantitative real-time PCR (qRT-PCR)

Total RNA was isolated from PDLSCs after 7 days of osteogenic induction using the SteadyPure Universal RNA Extraction Kit (AG, China), which included an on-column gDNA removal step. RNA concentration and purity were determined using a NanoDrop One spectrophotometer (Thermo Fisher Scientific, USA). cDNA was synthesized with HiScript^®^ III RT SuperMix (Vazyme, China). Quantitative PCR was performed using ChamQ Universal SYBR qPCR Master Mix (Vazyme, China) on a 7300 Sequence Detection System (Applied Biosystems, USA). The primer sequences are listed in Table S1.

### Western blot analysis

After 7 days of osteogenic induction, the PDLSCs were lysed on ice for 30 min using RIPA lysis buffer (Beyotime, China) supplemented with 1 mM phenylmethylsulfonyl fluoride (PMSF) and a protease inhibitor cocktail. The lysates were subsequently centrifuged at 12,000 × g for 10 min at 4 °C, and the resulting supernatants were collected. The protein concentration was quantified using a BCA assay (Beyotime, China). Protein samples were denatured by boiling in 5× SDS-PAGE loading buffer (Beyotime, China) at 95 °C for 5 min. Equal amounts of protein (20–40 µg per lane) were separated by electrophoresis on Bis-Tris precast gels (ACE, China) and then transferred onto polyvinylidene fluoride (PVDF) membranes (Millipore, USA) using a semi-dry transfer system (Thermo Fisher, USA). After blocking with 5% BSA for 1 h, the membranes were incubated overnight at 4 °C with the following primary antibodies: anti-AMPK (Cell Signaling Technology, Cat#: 2532, 1:1000), anti-p-AMPK (Cell Signaling Technology, Cat#: 2535, 1:1000), anti-mTOR (Cell Signaling Technology, Cat#: 2983, 1:1000), anti-p-mTOR (Cell Signaling Technology, Cat#: 5536, 1:1000), anti-Collagen I (Proteintech, Cat#: 14695-1-AP, 1:500), anti-OPN (ABclonal, Cat#: A19092, 1:500), anti-ALP (ABclonal, Cat#: A0514, 1:500), anti-Runx2 (Cell Signaling Technology, Cat#: 12556, 1:500), anti-P62 (Proteintech, Cat#: 18420-1-AP, 1:1000), anti-Beclin1 (Proteintech, Cat#: 11306-1-AP, 1:1000), anti-LC3 (Proteintech, Cat#: 14600-1-AP, 1:1000), anti-DAP1 (MedChemExpress, Cat#: YA2641, 1:200) and anti-β-actin (Affinity, Cat#: AF7018, 1:5000). After washing with TBST, the membranes were incubated with horseradish peroxidase (HRP)-conjugated goat anti-mouse (Bioworld, Cat#: BS22356, 1:50,000) or rabbit (Proteintech, Cat#: BS22357, 1:50,000) secondary antibody for 1 h at room temperature. The protein bands were visualized using an enhanced chemiluminescence (ECL) substrate kit (ABclonal, China) and imaged with a chemiluminescence imaging system.

### Transcriptome sequencing analysis

In this study, transcriptomic analysis was performed on PDLSCs isolated from the inferior alveolar nerve transection group and the sham-operated group (with three independent biological replicates per group). Total RNA was extracted using TRIzol reagent (Thermo Fisher Scientific, USA). RNA-seq libraries were prepared using the VAHTS Universal V5 RNA-seq Library Prep Kit (Illumina, USA) and sequenced on an Illumina NovaSeq 6000 platform (Vazyme, USA) to generate 150 bp paired-end reads (achieving a minimum depth of 40 million clean reads per sample). The quality control of the raw reads was performed using fastp. The resulting high-quality clean reads were subsequently aligned to the Rattus norvegicus reference genome (rn6) using HISAT2. Gene expression was quantified using HTSeq-count. Potential batch effects were assessed and corrected using the ComBat algorithm from the sva R package where applicable. Differential expression analysis was performed with DESeq2. Principal component analysis (PCA) was performed to visualize overall gene expression variation and assess inter-group reproducibility. Differentially expressed genes (DEGs) were visualized via hierarchical clustering and radar plots. Functional enrichment analyses were conducted using the GO, KEGG, Reactome and WikiPathways databases (R v3.2.0). The results were visualized using bar, chord, and bubble plots. Transcriptome sequencing and analysis were performed by OE Biotech Co., Ltd. (Shanghai, China).

### Transmission electron microscopy (TEM)

To observe autophagic structures, PDLSCs were fixed with 2.5% glutaraldehyde in 0.1 M phosphate buffer (pH 7.4) for 2**–**4 h at 4 °C following 24 h of osteogenic induction. The samples were then post-fixed with 1% osmium tetroxide for 1 h, dehydrated through a graded ethanol series, embedded in epoxy resin, and sectioned into 70–90 nm ultrathin sections. The sections were double-stained with uranyl acetate and lead citrate and examined using a Hitachi HT-7800 transmission electron microscope (Hitachi, Japan) to identify autophagosomes and autolysosomes.

For SC-EV characterization, a 10 µL aliquot of the vesicle suspension was adsorbed onto a formvar-carbon coated copper grid for 10 min. After excess liquid was removed, the grid was negatively stained with 2% phosphotungstic acid (pH 7.0) for 1 min, air-dried, and then imaged at 120 kV.

### mRFP-EGFP-LC3 puncta assay for autophagic flux

To monitor autophagic flux, PDLSCs were seeded in confocal dishes and cultured in osteogenic medium for 24 h. The cells were then transduced with mRFP-EGFP-LC3 adenovirus (MOI = 200; Hanbio, China) for 24 h. After fixation with 4% paraformaldehyde (PFA), the fluorescent puncta were imaged using a laser scanning confocal microscope (Nikon, Japan). Yellow puncta (EGFP^+^/mRFP^+^) represent autophagosomes, whereas red-only puncta (mRFP^+^) indicate autolysosomes.

### Co-culture experiments

To evaluate the effect of Schwann cells on PDLSC osteogenesis, RSC96 cells and PDLSCs were first co-cultured in a non-contact Transwell system (0.4 μm pore size) in osteogenic medium for 7 or 21 days. After fixation with 4% paraformaldehyde, ALP staining was performed on day 7 and ARS staining on day 21. To further investigate the paracrine mechanism, PDLSCs were treated with SC-derived extracellular vesicles or Schwann cell-conditioned medium (CM). For CM preparation, RSC96 cells were cultured for 48 h, and the collected medium was centrifuged to remove cells and debris. PDLSCs were divided into four groups: control, SC-EVs, CM, and CM + GW4869. In the CM + GW4869 group, RSC96 cells were pretreated with 10 µM GW4869 (Sigma, USA), an inhibitor of exosome/EV secretion, for 48 h before CM collection.

### Isolation, characterization, and cellular uptake of SC-EVs

SC-EVs were isolated from the RSC96 rat Schwann cell line supernatants via differential ultracentrifugation. Briefly, to prepare exosome-depleted fetal bovine serum (FBS), complete FBS was subjected to ultracentrifugation at 120,000×g for 14 h at 4 °C. The medium was sequentially centrifuged at 2,000×g and 10,000×g to remove cell debris, the vesicles were pelleted by ultracentrifugation at 110,000×g for 70 min. The resulting pellet was then resuspended in an adequate volume of sterile PBS and subjected to a second ultracentrifugation under identical conditions (110,000×g, 70 min, 4 °C). The final pellet was resuspended in PBS, quantified using a BCA assay, and stored at **−** 80 °C. The size distribution and concentration of SC-EVs were assessed using nanoparticle tracking analysis (NTA) with a ZetaView system (Particle Metrix, Germany). Western blotting confirmed the presence of the exosomal markers CD63 (Proteintech, Cat#: 25682-1-AP, 1:200), CD9 (Abcam, Cat#: ab92726, 1:1000), CD81 (Abcam, Cat#: ab109201, 1:1000), and TSG101 (Abcam, Cat#: ab125011, 1:1000). For uptake assays, SC-EVs were labeled with PKH26 (Sigma, USA), repelleted, and co-cultured with PDLSCs for 12 h. Internalization was visualized by confocal microscopy following fixation and DAPI counterstaining. All EV preparations used for comparative experiments were isolated using the same protocol and normalized by protein content. Based on the CCK-8 proliferation assay, 50 µg/mL was selected as the working concentration for subsequent experiments.

### Small RNA sequencing analysis

Total RNA was extracted using the mirVana miRNA Isolation Kit (Ambion, USA), the RNA concentration was measured using a NanoDrop 2000 spectrophotometer (Thermo Fisher Scientific, USA), and RNA integrity was assessed with an Agilent 2100 Bioanalyzer (Agilent, USA). Libraries were constructed from 1 µg of total RNA using the NEBNext Small RNA Library Prep Set (NEB, USA) through adapter ligation, reverse transcription, and PCR amplification (140–160 bp fragments). Library quality and size distribution were validated using the Agilent 2100 Bioanalyzer. Sequencing was performed on an Illumina NovaSeq 6000 platform (Illumina, USA) with 50 bp paired-end reads. Raw reads were filtered to remove low-quality sequences, adapter contaminants, and fragments outside 15–41 nt. Novel miRNAs were predicted using miRDeep2. Targets were predicted with miRanda and analyzed via GO/KEGG enrichment based on hypergeometric distribution. Small RNA sequencing and analysis were conducted by OE Biotech Co., Ltd. (Shanghai, China).

### miRNA extraction and stem-loop cDNA synthesis

miRNAs were isolated from SC-EVs using RNA Isolater lysis buffer (Vazyme, China). Following chloroform-ethanol phase separation, the samples were purified with a miPure RNA spin column, sequentially washed, and eluted with RNase-free water. The stem-loop primers used are listed in Table S1. HiScript II Reverse Transcriptase (Vazyme, China) was used to synthesize cDNA via gDNA removal and reverse transcription reactions. Products were stored at **−** 20 °C.

### Functional studies using miR-210-5p mimic and inhibitor

To investigate the functional role of rno-miR-210-5p, PDLSCs were transfected with a rno-miR-210-5p mimic (to overexpress the miRNA), a rno-miR-210-5p inhibitor (to knock down its function), or their respective negative controls (all from GenePharma, China). PDLSCs were seeded in 24-well plates 24 h prior to transfection. A transfection complex was prepared by mixing 15 pmol siRNA mimic/inhibitor with 8.5 µL of buffer, followed by the addition of 3 µL of plus transfection reagent. The complex was uniformly applied to the cell cultures, gently mixed, and further incubated.

### Prediction and dual-luciferase validation of rno-miR-210-5p targets

The potential targeting of the death-associated protein 1 (*DAP1*) 3’ untranslated region (3’UTR) by rno-miR-210-5p was predicted using the TargetScan algorithm. Luciferase reporter vectors (GenePharma, China) containing either the wild-type (WT) *DAP1* 3’UTR or a mutant (MUT) version with altered binding sites were co-transfected with the rno-miR-210-5p mimic or negative control into PDLSCs. After 24–48 h of culture, the cells were lysed and subjected to measuring firefly/Renilla luciferase activity using the Dual‑Luciferase Reporter Assay System.

### Statistical analysis

Data analysis and visualization were performed using GraphPad Prism 8.0, with continuous variables presented as the mean ± standard deviation (mean ± SD). Normality and homogeneity of variance were assessed using the Shapiro–Wilk test and Levene’s test, respectively. For comparisons between two groups, an unpaired (independent) t-test was used when data were normally distributed with equal variances, Welch’s t-test was used when variances were unequal, and the Mann–Whitney U test was used for non-normally distributed data. For comparisons among multiple groups, one-way ANOVA followed by Tukey’s or Šidák’s post hoc test was used for normally distributed data, and the Kruskal–Wallis test was used for non-normally distributed data. Pearson correlation analysis was used to evaluate the relationship between S100-positive cell density and bone microstructural parameters. The statistical methods and sample sizes for individual experiments are indicated in the corresponding figure legends. *P* < 0.05 was considered statistically significant.

## Results

### Temporal dynamics of bone microarchitecture and functional impairment of PDLSCs following denervation

**Fig. 1:**
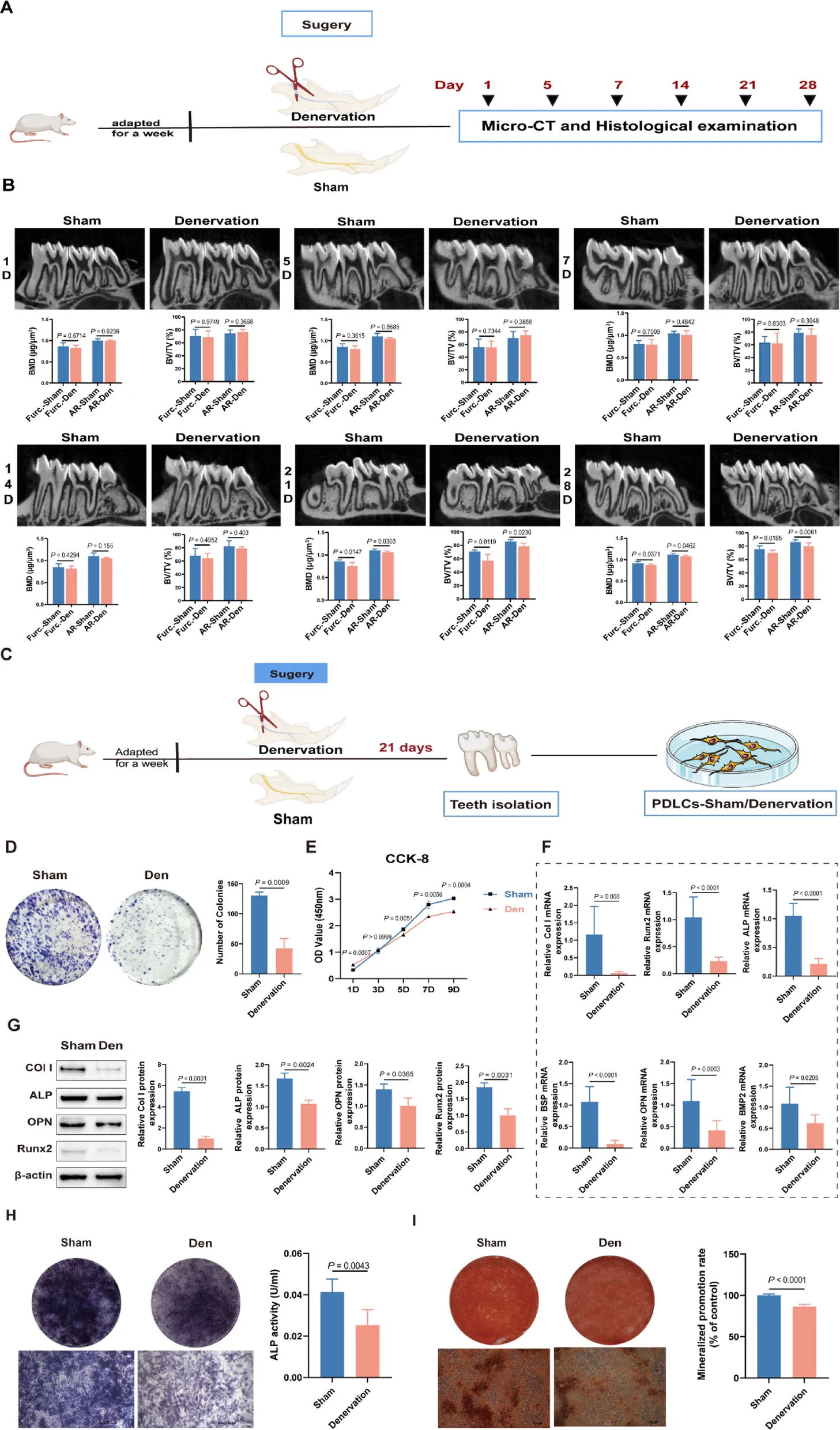
Progressive bone deterioration and PDLSC dysfunction following IAN transection. (**A**) Establishment of the rat inferior alveolar nerve transection model. (**B**) Micro-CT of the mandible at 1–28 days post-nerve transection. (**C**) Flowchart of cell experiment design. (**D**) Colony-forming ability of PDLSCs after nerve transection. (**E**) Cell proliferation assessed by CCK-8 assay. (**F**) Effect of denervation on the expression of osteogenesis-related genes in PDLSCs. (**G**) Effect of denervation on the expression of osteogenesis-related proteins in PDLSCs. (**H**) ALP activity in PDLSCs after denervation. (**I**) Mineralized nodule formation in PDLSCs after denervation. For panel B, *n* = 6 animals per group. For panels D-I, *n* = 3 independent biological replicates. Statistical analyses were performed as follows: panel B and D unpaired t-test; panel E, two-way ANOVA followed by Šidák’s multiple comparisons test; panel F, Mann**–**Whitney U test was used for ALP and BSP, while Welch’s t-test was used for the other comparisons; panel G, unpaired t-test; panel H, Mann**–**Whitney U test; panel I, unpaired t-test. Data are presented as mean ± SD

To characterize the progression of periodontal degeneration subsequent to inferior alveolar nerve (IAN) transection, we conducted longitudinal assessments of mandibular bone microstructure at 1, 5, 7, 14, 21, and 28 days post-surgery (Fig. 1A). Micro-CT analysis revealed decreases in bone volume fraction (BV/TV) and bone mineral density (BMD) in the molar furcation and alveolar ridge regions at 21 and 28 days following nerve transection (Fig. 1B). After observing bone architectural changes in periodontal tissues following IAN transection, we further investigated the corresponding effects on rPDLSCs at the cellular level. PDLSCs were isolated and cultured from the periodontal ligaments of rats 21 days after nerve transection. Multilineage differentiation assays showed the mesenchymal stem cell (MSC) properties of cells isolated from both groups, as evidenced by their osteogenic and adipogenic differentiation potential (Fig. 1I and S1A). Flow cytometric profiling further revealed the preservation of canonical MSC surface markers (CD90, CD29, CD105, and STRO-1) with minimal hematopoietic contamination (CD11b and CD45), suggesting that denervation does not substantially alter the immunophenotypic identity of PDLSCs (Fig. S1C and S1B). However, functional characterization revealed marked discrepancies between the experimental groups. CFU assays and CCK-8 proliferation studies revealed significantly attenuated clonogenic capacity and proliferation kinetics in denervated PDLSCs compared with those in controls (Fig. 1D and E). Osteogenic differentiation capacity was also impaired, as reflected by the decreased expression of osteogenic transcripts (ALP, BSP, BMP2, Col I, OPN, and Runx2) and proteins (ALP, Col I, OPN, and Runx2) (Fig. 1F and G), together with decreased alkaline phosphatase (ALP) activity on day 7 (Fig. 1H). Extracellular matrix mineralization, assessed by Alizarin Red S (Fig. 1I) and Von Kossa staining (Fig. S1D) on day 21, appeared reduced in denervated PDLSCs, consistent with impaired late-stage osteogenic capacity. Quantitative analyses further showed decreased matrix deposition. Collectively, these data indicate that IAN transection is associated with progressive deterioration of bone microarchitecture and impaired PDLSC function.

### Correlations between Schwann cell depletion and periodontal bone alterations

**Fig. 2:**
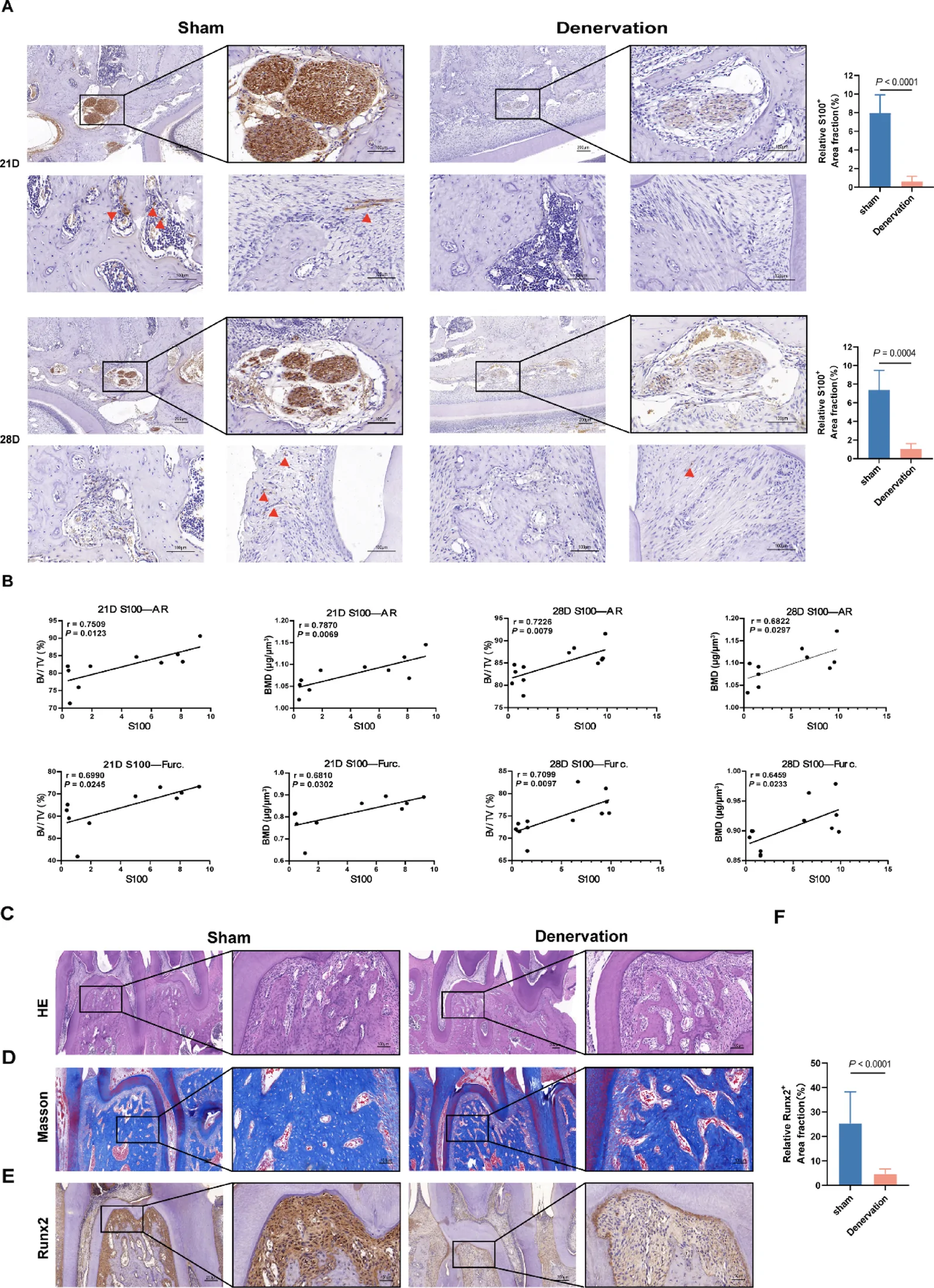
Schwann cell depletion correlates with periodontal bone loss after denervation. (**A**) Immunohistochemical staining of S100 in the periodontal ligament at 21 and 28 days post-operation. (**B**) Pearson correlation analysis between S100-positive cell density and bone microstructural parameters (BV/TV and BMD) at days 21 and 28 (Furc.: Furcation; AR: alveolar ridge). (**C**) H&E staining of rat periodontal tissues at 21 days post-operation. (**D**) Masson’s trichrome staining of rat periodontal tissues at 21 days post-operation. Scale bars: 200 μm and 100 μm. (**E**) Immunohistochemical staining of Runx2 in periodontal tissues at 21 days post-operation. Scale bars: 50 μm and 200 μm. (**F**) Semi-quantitative analysis of Runx2 expression. *n* = 6 per group. Statistical analysis: Panel A, Mann–Whitney U test for the 21-day and unpaired t-test for the 28-day; panel B, Pearson correlation analysis; panel F, Mann–Whitney U test. Data are presented as mean ± SD

Building on the above findings, we next assessed the status of Schwann cells in periodontal tissue to determine whether they changed after IAN transection. Immunohistochemical staining for S100, a canonical Schwann cell marker, showed that Schwann cell populations in periodontal tissue were generally maintained from days 1 to 14 following IAN transection. Although staining intensity and cell counts exhibited minor fluctuations over time, no statistically significant differences were observed between the two groups at any examined time point (Fig. S2A-D). On days 21 and 28, both the number of Schwann cells and the intensity of S100 immunoreactivity were markedly reduced compared with those in the sham control group (Fig. 2A). These trends are consistent with the dynamic changes in SCs after sciatic nerve transection [21] and the SC depletion phenomenon following IAN injury reported previously [22]. Pearson correlation analysis was conducted to examine the relationships between the expression of the SC marker S100 and bone microstructural parameters (BV/TV and BMD) at 21 and 28 days post-nerve transection. The analysis revealed a significant positive correlation between the number of SCs and both BV/TV and BMD (Fig. 2B). Concurrent histological examination using H&E staining revealed a reduction in bone and osteoid tissue, accompanied by relative expansion of the marrow cavity, consistent with the micro-CT findings (Fig. 2C). Masson’s trichrome staining showed a trend toward a sparser collagen fiber network within the alveolar bone (Fig. 2D). Additionally, immunohistochemical analysis revealed significantly decreased Runx2 expression in periodontal tissues (Fig. 2E and F). Taken together, these findings indicate that IAN transection induces progressive Schwann cell depletion in periodontal tissue, which temporally parallels bone structural deterioration and is correlated with reduced bone parameters.

### The neural microenvironment is associated with osteogenic differentiation of PDLSCs via the AMPK-mTOR-autophagy axis

**Fig. 3:**
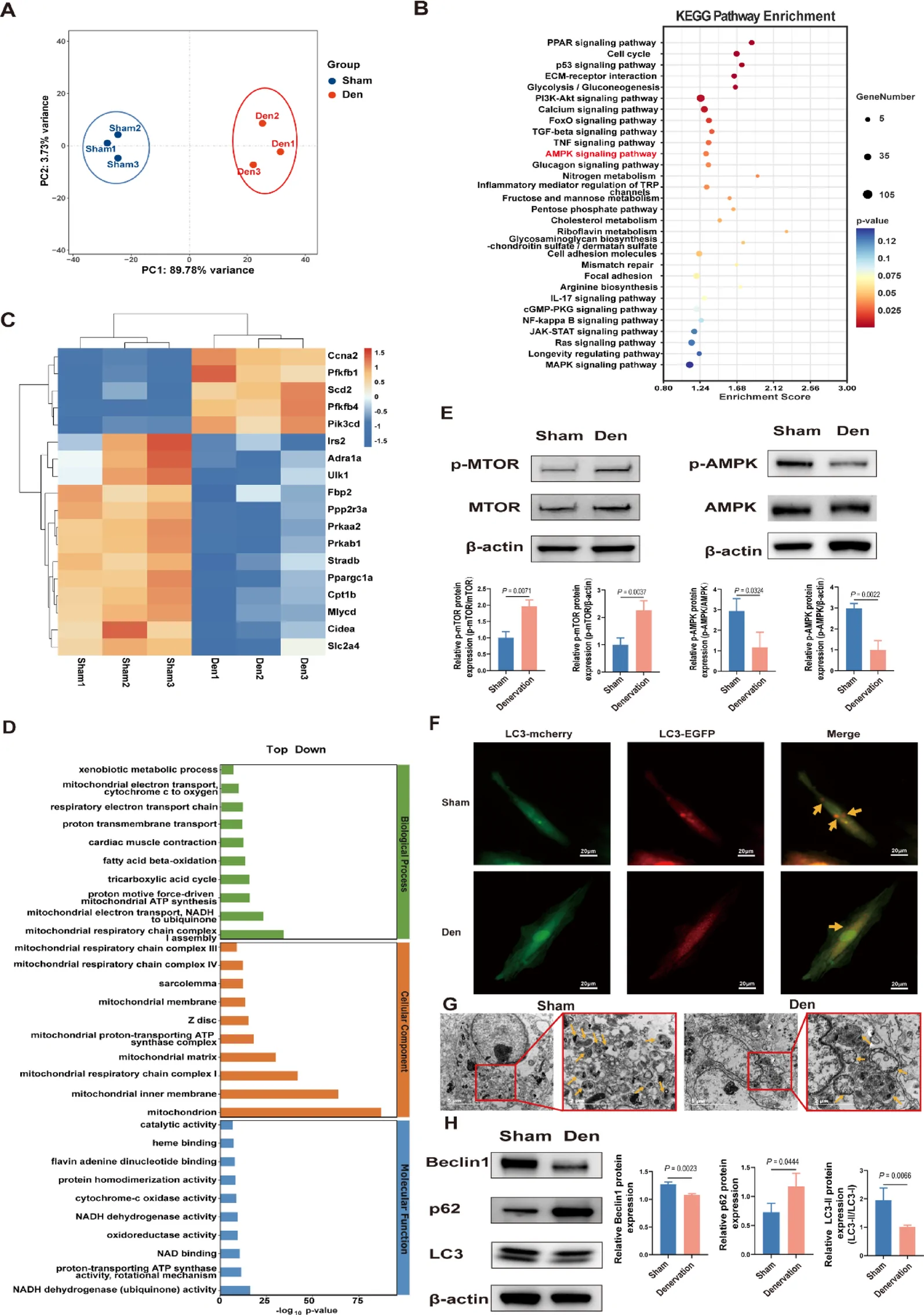
The neural microenvironment influences the osteogenic differentiation of PDLSCs by modulating the AMPK-mTOR-autophagy axis. (**A**) Principal component analysis (PCA) plot of the transcriptomes of PDLSCs from the denervated and sham-operated groups. (**B**) KEGG enrichment analysis of the transcriptomes of PDLSCs from the denervated and sham-operated groups. (**C**) Heatmap of AMPK signaling pathway components from the transcriptomic data of PDLSCs in the denervated and sham-operated groups. (**D**) Gene Ontology (GO) enrichment analysis of the transcriptomes of PDLSCs from the denervated and sham-operated groups. (**E**) Effect of denervation on the expression of AMPK-mTOR pathway-related proteins in PDLSCs. (**F**) Effect of denervation on LC3-autophagic flux in PDLSCs. Scale bars: 20 μm. Yellow arrows: Autophagosomes; red arrows: Autolysosomes. (**G**) Transmission electron microscopy (TEM) analysis of the effect of denervation on autophagy in PDLSCs. Yellow arrows indicate autophagosomes and autolysosomes. Scale bars: 2 μm and 5 μm. (**H**) Effect of denervation on the expression of autophagy-related proteins in PDLSCs. For panels E-H, *n* = 3 independent biological replicates. Statistical analysis: panels E and H, unpaired t-test. Data are presented as mean ± SD

To investigate the molecular mechanisms through which IAN transection regulates PDLSCs, we performed RNA sequencing (RNA-seq) analysis on PDLSCs isolated from the denervation and sham groups. RNA-seq analysis revealed that IAN transection significantly altered the gene expression profile of PDLSCs (Fig. 3A), with 2428 genes significantly upregulated and 2309 genes significantly downregulated in the denervation group (Fig. S3A). KEGG pathway enrichment analysis indicated that IAN transection was associated with AMPK signaling in PDLSCs (Fig. 3B). Gene Ontology (GO) enrichment analysis revealed that the differentially expressed genes were significantly enriched in biological processes and molecular functions related to mitochondrial respiratory chain complex I, NADH dehydrogenase activity and proton motive force-driven mitochondrial ATP synthesis (Fig. 3D). These findings suggest that the denervation microenvironment may be associated with changes in gene expression related to cellular energy metabolism. This imbalance could suppress the expression and kinase activity of key subunits such as AMPKα1, thus exacerbating the dysregulation of the AMPK/mTOR signaling axis. A heatmap visualizing the expression of core components of the AMPK pathway revealed that, compared with the sham group, the denervation group exhibited significant downregulation of genes encoding key AMPK complex components, including the catalytic subunit Prkaa2, regulatory subunit Prkab1, and the upstream kinase scaffold Stradb. Concomitantly, key energy metabolism genes, including Ppargc1a, Cpt1b and Slc2a4, were coordinately downregulated, reflecting transcriptional changes consistent with impaired fatty acid oxidation and glucose transport in the denervated microenvironment (Fig. 3C). Furthermore, the downregulation of Ulk1, a critical initiator of autophagy, in the IAN-transected group suggested that autophagic activity in PDLSCs may be affected (Fig. 3C). To validate these transcriptomic findings, the expression levels of molecules in the AMPK-mTOR pathway were assessed by qPCR and Western blotting. The results showed that IAN transection significantly inhibited AMPK activity but activated mTOR activity (Fig. 3E and Fig. S3B). Notably, the phosphorylation levels of AMPK and mTOR were significantly negatively correlated, indicating a potential antagonistic regulatory relationship between them under denervated conditions. By integrating the transcriptomic and biochemical data, these findings suggest that IAN transection is associated with both SC depletion and alterations in the AMPK-mTOR pathway, which may be linked to the impaired osteogenic differentiation potential of PDLSCs. AMPK is a key regulator of autophagy initiation and can influence osteogenic differentiation by modulating autophagy [23]. Based on the transcriptomic data suggesting the downregulation of the autophagy initiator Ulk1 after denervation, this study further investigated the effect of denervation on the autophagic activity of PDLSCs. Transfection of PDLSCs with the mCherry-EGFP-LC3 autophagy reporter revealed that the denervated group exhibited reduced autophagic flux (Fig. 3F). Consistently, TEM revealed a reduction in the number of autophagosomes and autolysosomes in the denervation group (Fig. 3G). In line with these findings, the results of the qPCR and Western blotting indicated impaired autophagic activity in the denervation group (Fig. 3H and S3C). These findings suggest a potential association between nerve transection and reduced Schwann cell numbers, as well as suppression of the AMPK-mTOR signaling axis in PDLSCs, which may be related to impaired autophagic activity.

### Regulatory role of SC-EVs in the osteogenic differentiation of PDLSCs

**Fig. 4:**
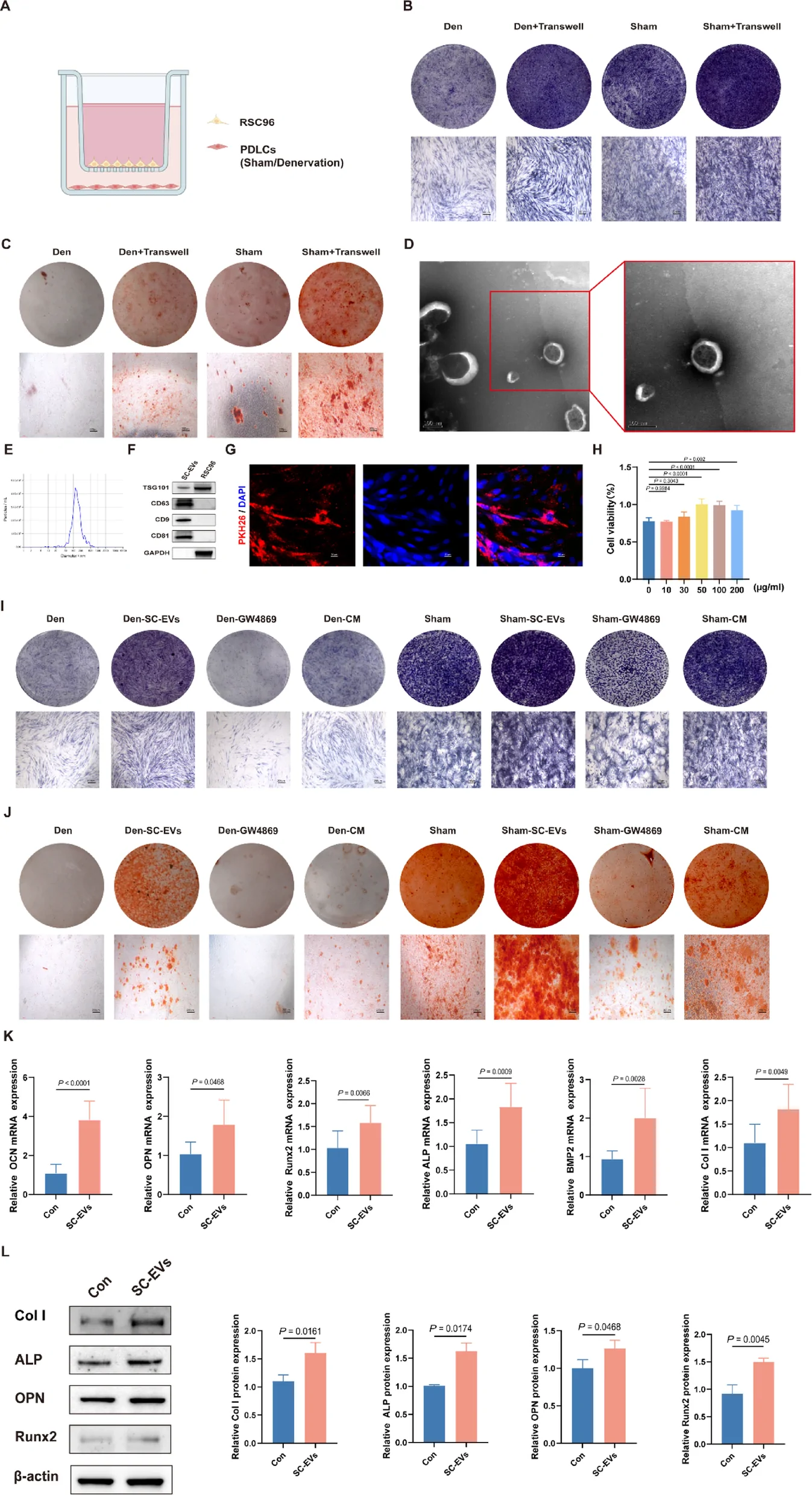
Regulatory role of SC-EVs on the osteogenic differentiation of PDLSCs. (**A**) Schematic diagram of the co-culture system between RSC96 and PDLSCs. (**B**) Effect of RSC96 on ALP activity in PDLSCs. (**C**) Effect of RSC96 on mineralized nodule formation in PDLSCs. (**D**) TEM image of isolated SC-EVs showing the characteristic cup-shaped morphology. Scale bar: 100 nm. (**E**) NTA of SC-EVs. (**F**) Western blot analysis of SC-EV markers TSG101, CD9, CD63, and CD81. (**G**) Cellular uptake of SC-EVs by PDLSCs. Red fluorescence: PKH26; blue fluorescence: DAPI. Scale bar: 20 μm. (**H**) Effect of SC-EVs on the proliferation of PDLSCs. (**I**) Effect of SC-EVs on ALP activity in PDLSCs. (**J**) Effect of SC-EVs on mineralized nodule formation in PDLSCs. (**K**) Effect of SC-EVs on the expression of osteogenesis-related genes in PDLSCs. (**L**) Effect of SC-EVs on the expression of osteogenesis-related proteins in PDLSCs. *n* = 3 independent biological replicates. Statistical analysis: panels H, ordinary one-way ANOVA; panels K-L, unpaired t-test. Data are presented as mean ± SD

As the most abundant glial cells in the peripheral nervous system, SCs not only support nerve conduction and axonal regeneration but also drive cross-tissue repair by remodeling the microenvironment and regulating key signaling pathways [7]. Histological studies further revealed that IAN transection leads to Schwann cell depletion and subsequent impairment of periodontal tissues. To investigate the effect of Schwann cells on PDLSCs, we established a transwell co-culture system under osteogenic induction conditions (Fig. 4A). ALP and ARS staining revealed that the SCs promoted the osteogenic differentiation of the PDLSCs (Fig. 4B, C). Given the pivotal role of EVs in intercellular communication, we hypothesized that SC-EVs may mediate the pro-osteogenic effects of SCs on PDLSCs. SC-EVs were isolated from the rat Schwann cell line RSC96. Transmission electron microscopy, nanoparticle tracking analysis, and Western blotting analysis showed that the isolated SC-EVs exhibited a characteristic cup-shaped morphology, an average size of approximately 110 nm, and high expression of the characteristic markers CD9, CD63, CD81, and TSG101 (Fig. 4D, E, F). PKH26 fluorescent labeling revealed the efficient internalization of SC-EVs by PDLSCs (Fig. 4G). CCK-8 assays revealed that SC-EVs promoted PDLSC proliferation in a dose-dependent manner (50–200 µg/mL), with 50 µg/mL identified as the minimal effective concentration for subsequent experiments (Fig. 4H). To investigate the potential contribution of SC-EVs, we treated PDLSCs with SC-EVs, RSC96-conditioned medium (CM), and CM from RSC96 pretreated with GW4869 (CM+GW4869). After osteogenic induction, SC-EV treatment enhanced osteogenic differentiation of PDLSCs, as evidenced by increased ALP staining and matrix mineralization (ARS), whereas GW4869 treatment attenuated the pro-osteogenic effect of SC-CM (Fig. 4I, J). At the molecular level, qPCR and Western blotting showed that SC-EVs increased the expression of key osteogenic marker genes and proteins (Fig. 4K, L). Taken together, these results suggest a potential role for SC-EVs in promoting the osteogenic differentiation of PDLSCs.

### Effects of SC-EVs on the AMPK-mTOR-autophagy axis in PDLSCs

**Fig. 5:**
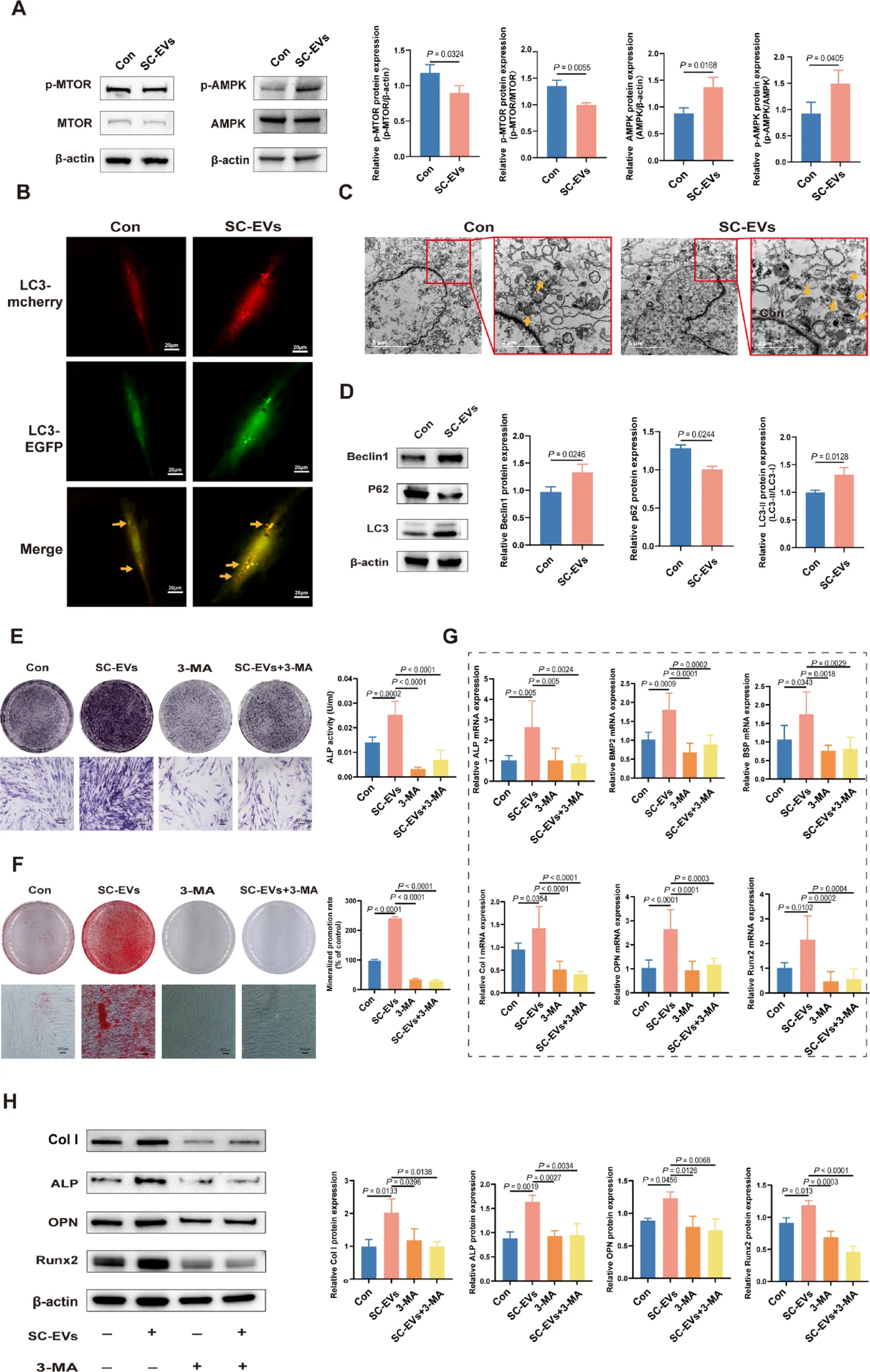
Effect of SC-EVs on the AMPK-mTOR-autophagy axis in PDLSCs. (**A**) Effect of SC-EVs on the expression of AMPK-mTOR pathway-related proteins in PDLSCs. (**B**) Effect of SC-EVs on LC3-autophagic flux in PDLSCs. Scale bars: 20 μm. Yellow arrows: Autophagosomes; red arrows: Autolysosomes. (**C**) TEM analysis of the effect of SC-EVs on autophagy in PDLSCs. Yellow arrows indicate autophagosomes and autolysosomes. Scale bars: 2 μm and 5 μm. (**D**) Effect of SC-EVs on the expression of autophagy-related proteins in PDLSCs. (**E**) The autophagy inhibitor 3-MA attenuated the SC-EV-induced enhancement of ALP activity in PDLSCs. (**F**) 3-MA inhibited the SC-EV-promoted mineralized nodule formation in PDLSCs. (**G**) 3-MA suppressed the SC-EV-induced upregulation of osteogenesis-related genes in PDLSCs. (**H**) 3-MA counteracted the SC-EV-induced increase in osteogenesis-related protein expression in PDLSCs. *n* = 3 independent biological replicates. Statistical analysis: panels A and D, unpaired t-test; panels E-H, ordinary one-way ANOVA. Data are presented as mean ± SD

We next investigated whether the pro-osteogenic effect of SC-EVs involves the AMPK**-**mTOR-autophagy axis. PDLSCs treated with SC-EVs were analyzed by qRT-PCR and Western blotting. The results showed that SC-EVs significantly increased AMPK mRNA expression and suppressed mTOR mRNA levels, which were accompanied by elevated p-AMPK levels and reduced p-mTOR levels (Fig. 5A and Fig. S4B). Autophagy analysis revealed that SC-EVs enhanced autophagic flux, as indicated by an increase in mCherry-EGFP-LC3 fluorescent puncta (Fig. 5B) and a greater number of autophagosomes and autolysosomes under electron microscopy (Fig. 5C). Furthermore, qRT-PCR and Western blotting analyses revealed concomitant upregulation of autophagy-related genes (Beclin1 and Atg5) and downregulation of p62 (Fig. S4C), along with an increased LC3-II/LC3-I ratio and decreased p62 protein levels (Fig. 5D). These data indicate that SC-EVs can upregulate autophagic flux by modulating the AMPK-mTOR pathway. To investigate whether SC-EV-induced autophagy contributes to osteogenic enhancement, we treated cells with the autophagy inhibitor 3-methyladenine (3-MA). Strikingly, 3-MA treatment significantly attenuated the SC-EV-induced increase in osteogenic differentiation, reversing the increases in ALP activity (Fig. 5E), mineralized nodule formation (Fig. 5F), and osteogenic marker expression at both the mRNA and protein levels (Fig. 5G, H). These rescue experiments suggest that the pro-osteogenic effect of SC-EVs may be largely dependent on autophagy activation.

### SC-EVs influence the autophagy and osteogenic differentiation of PDLSCs via miR-210-5p

**Fig. 6:**
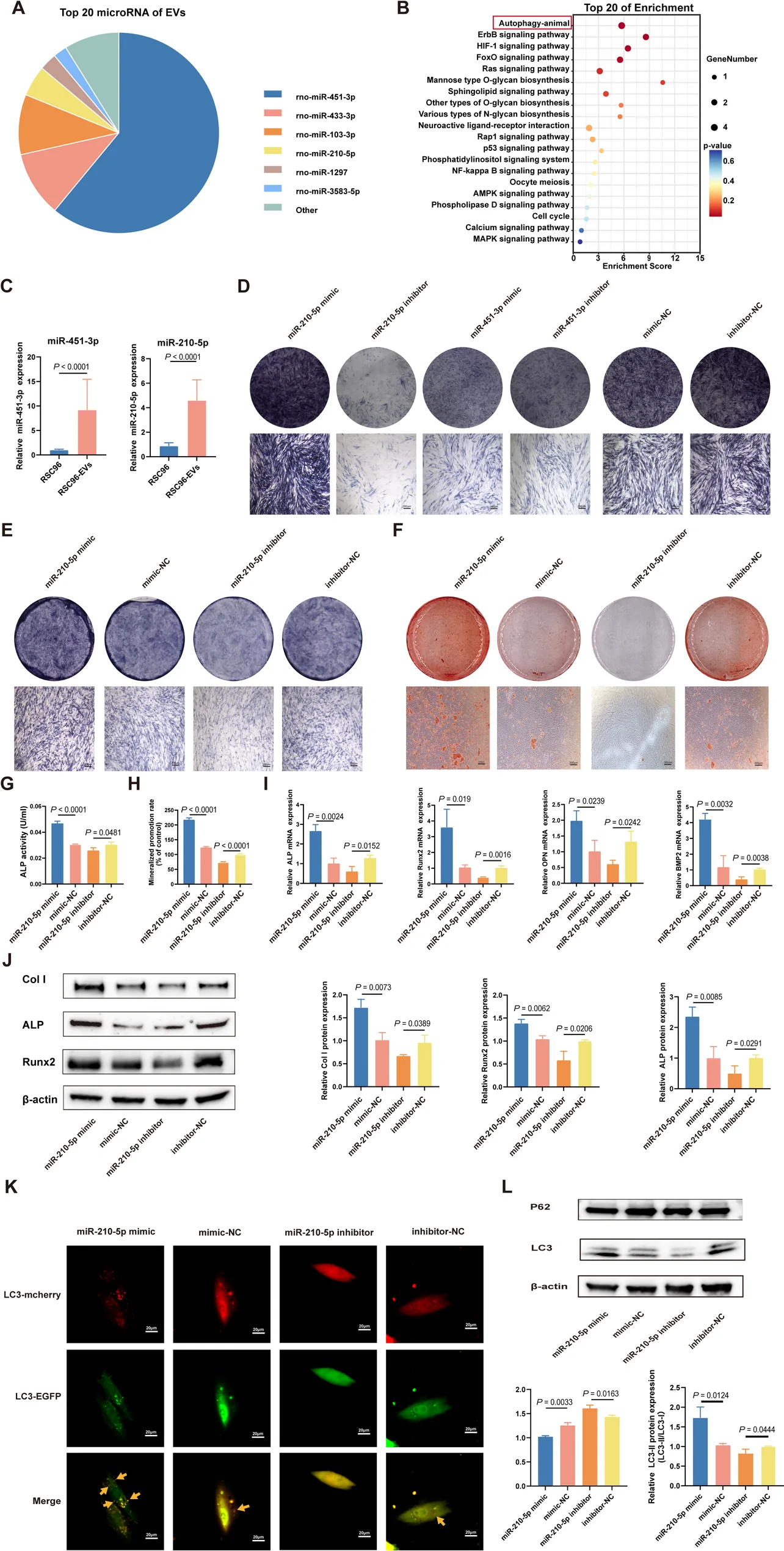
SC-EVs influence autophagy and osteogenic differentiation of PDLSCs via miR-210-5p. (**A**) miRNA enrichment profile in SC-EVs. (**B**) KEGG enrichment analysis of the identified miRNAs. (**C**) qPCR validation of selected miRNAs in SC-EVs. (**D**) Effect of miRNA mimics on ALP activity in PDLSCs. Scale bars: 200 μm. (**E**) miR-210-5p promotes ALP activity in PDLSCs. (**F**) miR-210-5p enhances mineralized nodule formation in PDLSCs. Scale bars: 200 μm. (**G**) Semi-quantitative analysis of ALP staining. (**H**) Semi-quantitative analysis of Alizarin Red S staining. (**I**) miR-210-5p upregulates the expression of osteogenesis-related genes in PDLSCs. (**J**) miR-210-5p upregulates the expression of osteogenesis-related proteins in PDLSCs. (**K**) Effect of miR-210-5p on LC3 autophagic flux in PDLSCs. Scale bars: 20 μm. Yellow arrows indicate autophagosomes. (**L**) miR-210-5p promotes the expression of autophagy-related proteins in PDLSCs. *n* = 3 independent biological replicates. Statistical analysis: panel C, Mann–Whitney U test and unpaired t-test; panel G-J and L, unpaired t-test. Data are presented as mean ± SD

Small RNA sequencing of SC-EVs revealed several highly expressed miRNAs, including rno-miR-451-3p, rno-miR-433-3p, rno-miR-103-3p, rno-miR-210-5p, rno-miR-1297 and rno-miR-3583-5p (Fig. 6A). KEGG pathway analysis revealed that these miRNAs were significantly enriched in the autophagy pathway (Fig. 6B). Among them, rno-miR-451-3p and rno-miR-210-5p have previously been implicated in the regulation of autophagy [24, 25]. qPCR results showed that the expression levels of rno-miR-451-3p and rno-miR-210-5p were significantly higher in SC-EVs than in RSC96 (Fig. 6C). A functional screen using miRNA mimics identified rno-miR-210-5p as a candidate that significantly increased ALP activity in PDLSCs under our experimental conditions, suggesting its potential role as a mediator of SC-EV-induced osteogenesis (Fig. 6D). To elucidate the mechanistic role of miR-210-5p, we transfected PDLSCs with either a rno-miR-210-5p mimic or inhibitor. The overexpression of miR-210-5p increased ALP activity (Fig. 6E, G), increased matrix mineralization (Fig. 6F, H), and upregulated the expression of key osteogenic genes and proteins (Fig. 6I, J). Concurrently, miR-210-5p overexpression was associated with enhanced autophagic activity, as evidenced by increased autophagic flux (Fig. 6K), a higher LC3-II/LC3-I ratio, elevated Beclin1 levels, reduced p62 protein expression (Fig. 6L), and corresponding changes in autophagy-related gene expression (Fig. S4D). These findings suggest that miR-210-5p may serve as a functional component of SC-EVs and contribute to their pro-osteogenic effects on PDLSCs, at least in part, through the modulation of autophagy.

### SC-EVs modulate PDLSCs by delivering miR-210-5p to target and silence DAP1

**Fig. 7:**
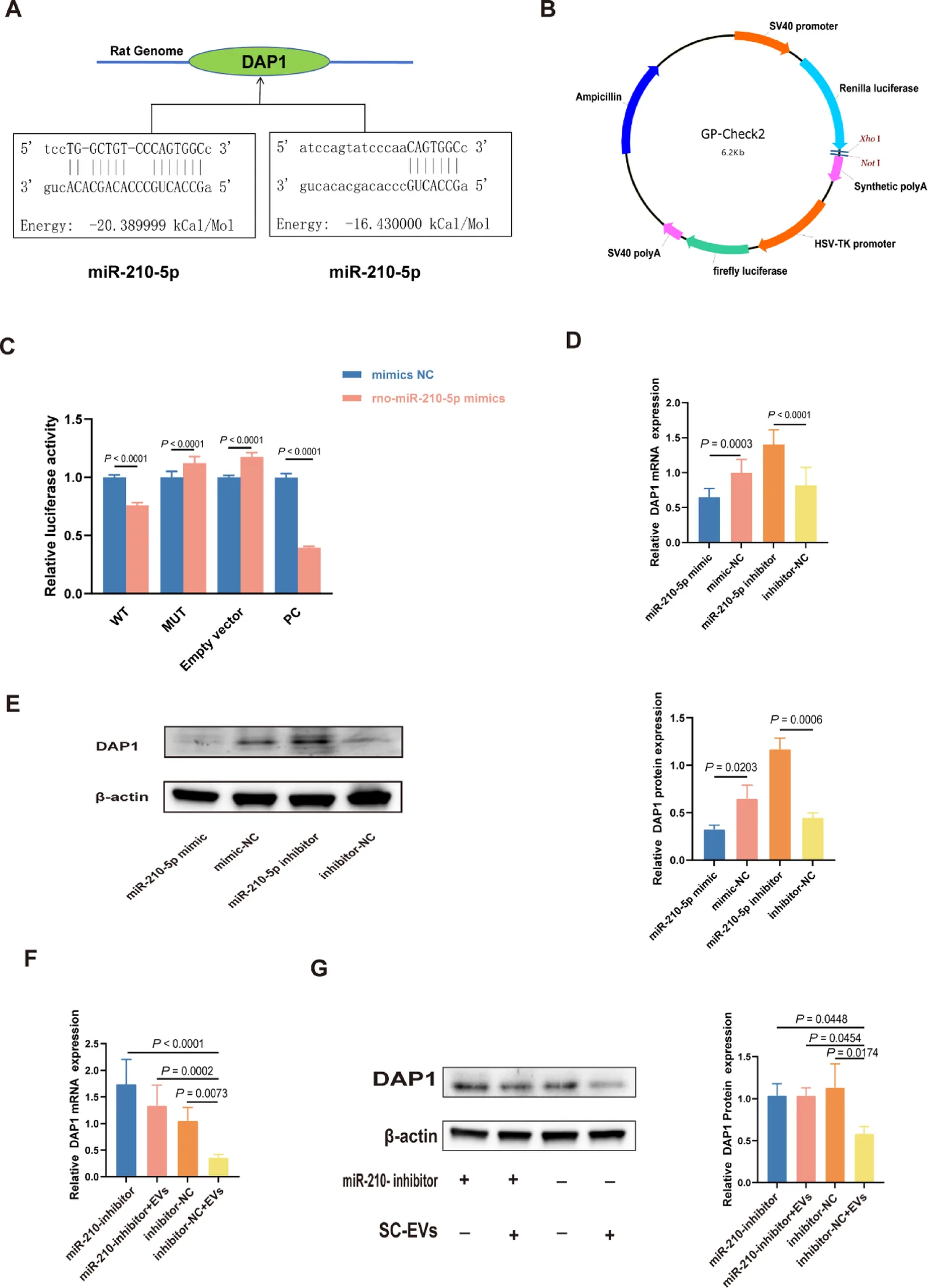
SC-EVs modulate PDLSCs by delivering miR-210-5p to target and silence DAP1. (**A**) Schematic diagram of the predicted binding site between miR-210-5p and the DAP1 gene. (**B**) Structure of the GP-CHECK-2 dual-luciferase miRNA target expression vector. (**C**) Binding of miR-210-5p to the DAP1 gene sequence as detected by dual-luciferase reporter assay. (**D**) Effect of miR-210-5p on DAP1 mRNA expression in PDLSCs. (**E**) Effect of miR-210-5p on DAP1 protein expression in PDLSCs. (**F**) Regulation of DAP1 mRNA expression in PDLSCs by SC-EVs via miR-210-5p. (**G**) Regulation of DAP1 protein expression in PDLSCs by SC-EVs via miR-210-5p. *n* = 3 independent biological replicates. panels C, Tukey’s multiple comparisons test; panel E, unpaired t-test; panels F and G, ordinary one-way ANOVA. Data are presented as mean ± SD

To elucidate the downstream mechanism through which miR-210-5p promotes autophagy, a potential target gene was predicted using the TargetScan database. The death-associated protein 1 (*DAP1*) gene was identified as a candidate, with a predicted binding site for miR-210-5p in its 3′ UTR (Fig. 7A). DAP1, a substrate of mTOR, directly participates in the negative regulation of autophagy [26]. A dual-luciferase reporter assay revealed that co-transfection with a miR-210-5p mimic significantly reduced luciferase activity of the wild-type *DAP1* 3′UTR reporter, whereas mutation of the predicted binding site abolished this effect (Fig. 7B, C), supporting the direct interaction between miR-210-5p and DAP1. Similarly, overexpression of miR-210-5p in PDLSCs significantly suppressed both DAP1 mRNA and protein levels, whereas inhibition of miR-210-5p had the opposite effect (Fig. 7D, E). To verify whether SC-EV-delivered miR-210-5p regulates DAP1 expression in PDLSCs, PDLSCs were treated with SC-EVs alone or in combination with a miR-210-5p inhibitor. qPCR analysis revealed that SC-EV treatment significantly reduced the DAP1 mRNA level, and this effect was largely reversed by co-treatment with the miR-210-5p inhibitor (Fig. 7F). Western blotting analysis showed a consistent decrease in DAP1 protein levels upon SC-EV treatment, which was reversed by the miR-210-5p inhibitor (Fig. 7G). These results indicate that SC-EVs deliver functional miR-210-5p to PDLSCs to suppress DAP1 expression. These results indicate that the miR-210-5p/DAP1 axis may be involved in SC-EV-associated autophagy activation and enhanced osteogenesis in PDLSCs.

### Effects of SC-EVs on the repair of denervated periodontal tissue defects

**Fig. 8:**
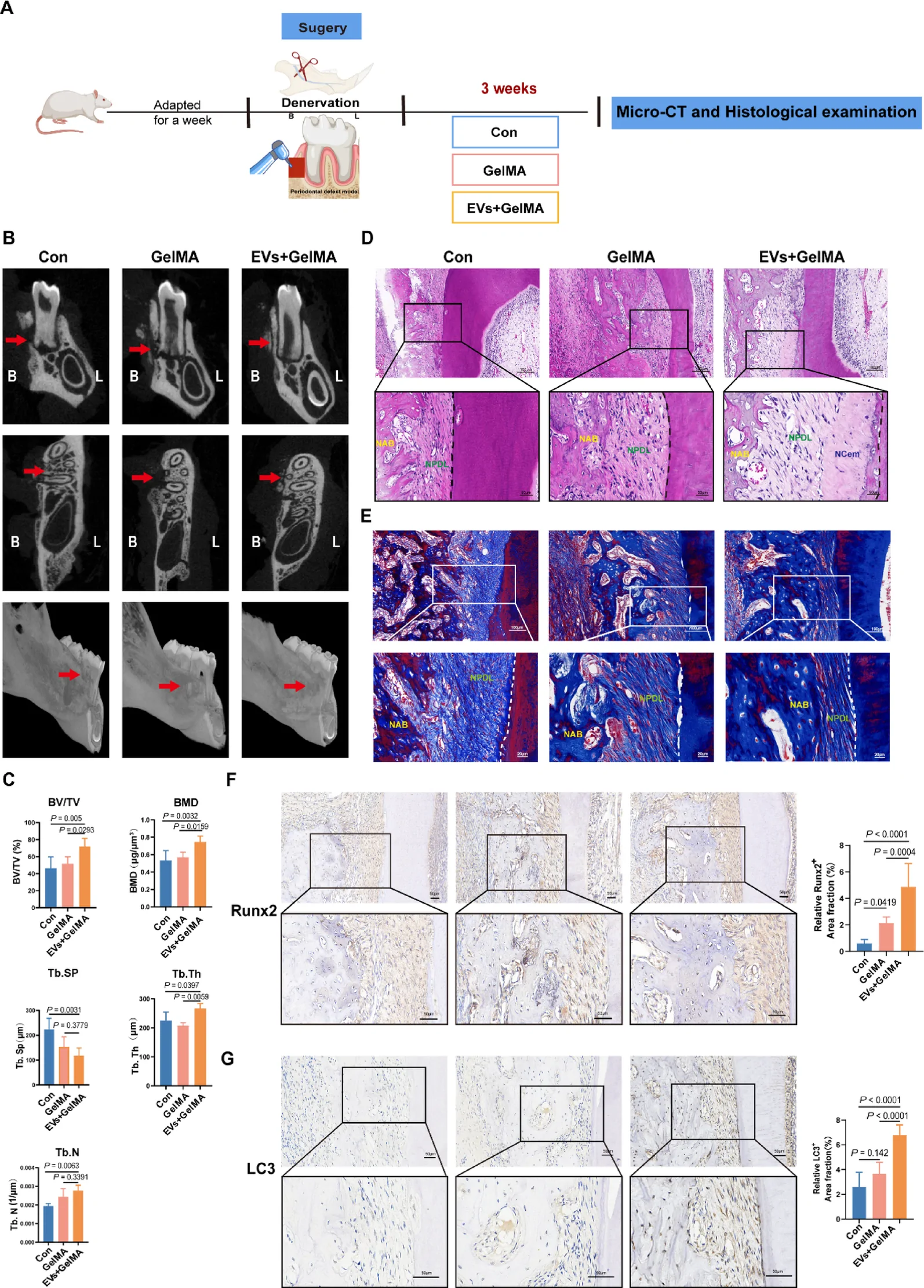
Effect of SC-EVs on the repair of denervated periodontal tissue defects. (**A**) Experimental design flowchart. (**B**) Micro-CT scans of mandibular defects from each group. (**C**) Quantitative analysis of bone parameters in the defect area. (BV/TV: bone volume fraction, BMD: bone mineral density, Tb.N: trabecular number, Tb.Th: trabecular thickness, Tb.Sp: trabecular separation.) (**D**) H&E staining of periodontal tissue defects. Scale bars: 100 μm and 20 μm. (**E**) Masson’s trichrome staining of periodontal tissue defects. Scale bars: 100 μm and 20 μm. (**F**) Immunohistochemical staining of Runx2 in periodontal tissue defects. Scale bars: 50 μm. (**G**) Immunohistochemical staining of LC3 in periodontal tissue defects. Scale bars: 50 μm. *n* = 6 animals per group. Statistical analysis: panels C and F–G, ordinary one-way ANOVA. Data are presented as mean ± SD

Finally, to validate our in vitro findings, we evaluated the therapeutic potential of SC-EVs for periodontal regeneration in a rat model in which IAN transection was combined with a periodontal defect. A photocurable GelMA hydrogel was used as a carrier for the local delivery and sustained release of SC-EVs. The rats were divided into three groups for comparison: a control group (no treatment), a GelMA-only group, and a GelMA group loaded with SC-EVs (EVs+GelMA) (Fig. 8A). At 21 days post-operation, micro-CT analysis revealed that, compared with both the control and GelMA-only groups, the EVs+GelMA group exhibited significantly greater bone regeneration, as evidenced by greater bone volume fraction (BV/TV) and bone mineral density (BMD) (Fig. 8B). A concurrent increase in trabecular thickness (Tb.Th) indicated the formation of denser bone structures. Furthermore, compared with those in the control group, Tb.Sp was significantly lower and Tb.N was significantly higher in the EVs+GelMA group (Fig. 8C). These quantitative micro-CT data suggest that SC-EVs delivered via the GelMA hydrogel significantly increase the quality and quantity of newly formed bone. H&E and Masson’s trichrome staining revealed histological features largely consistent with the micro-CT findings, suggesting improved tissue regeneration in the EVs+GelMA group. Specifically, the newly formed bone in the EVs+GelMA group exhibited denser trabeculae, a higher density of osteocytes, and a more organized cellular architecture, indicating advanced bone maturation. Moreover, the EVs+GelMA group displayed more orderly arranged periodontal ligament-like fibers, fewer inflammatory cells observed, and evidence of new cementum formation (Fig. 8D and E). Immunohistochemical analysis further revealed significantly increased expression of the osteogenic transcription factor Runx2 (Fig. 8F) and the autophagy marker LC3 (Fig. 8G) in the EVs+GelMA group. These in vivo data showed that local administration of SC-EVs improved repair-associated outcomes in rats with denervated periodontal defects, likely by concurrently enhancing osteogenic differentiation and autophagic activity.

## Discussion

In the present study, denervation-induced periodontal deterioration was evident by day 21 after IAN transection, as reflected by bone loss in the furcation and alveolar ridge regions together with reduced bone mineral density. PDLSCs, as a key mesenchymal stem cell population involved in periodontal homeostasis and repair-associated remodeling [27], also exhibited reduced proliferative capacity and impaired osteogenic differentiation potential under denervated conditions. In line with these changes, histological analysis showed decreased Runx2 expression in periodontal tissues, suggesting that the denervated microenvironment may be less supportive of osteogenic activity at this stage. Schwann cells, the main glial cells of the peripheral nervous system, develop from neural crest-derived Schwann cell precursors (SCPs) that retain broad developmental plasticity. This lineage is closely tied to craniofacial formation and remains functionally plastic in adults, thereby making Schwann cell-mediated regulation of multi-tissue regeneration plausible. Within the bone microenvironment, Schwann cells orchestrate pivotal regulatory functions, with their regenerative capacity being particularly important for mandibular defect repair [18, 28]. In this study, we next investigated whether the observed bone loss and PDLSC dysfunction were directly linked to SC depletion. Indeed, correlation analyses revealed that progressive SC depletion temporally paralleled the deterioration of alveolar bone microarchitecture, which is consistent with prior evidence that decreased SC numbers after IAN injury are associated with impaired mandibular repair through detrimental effects on skeletal stem cell function [22]. To functionally validate whether SCs could rescue PDLSCs from this denervated microenvironment, direct co-culture experiments revealed that SCs could enhanced osteogenic differentiation and restored the proliferative capacity of PDLSCs. These findings align with those of previous studies showing that SC-conditioned medium promotes osteoprogenitor mineralization through ERK signaling activation [29]. Collectively, these results suggest that SCs likely modulate PDLSC osteogenic activity via paracrine mechanisms, thus contributing to periodontal tissue homeostasis.

Although our co-culture findings support a paracrine mechanism for SC-mediated rescue of PDLSCs, the specific effector molecules involved remain to be identified. EVs are important mediators of intercellular communication through the transfer of bioactive cargo [30–32]. SC-EVs are increasingly recognized as key paracrine mediators of neuro-bone crosstalk, with reported roles in promoting osteogenic differentiation through signaling pathways such as TGFβ-Smad/MAPK and functional miRNA delivery [10, 17, 33]. Engineered EV-based biomimetic periosteum has also been shown to promote bone repair [34]. In our study, treatment with the exosome secretion inhibitor GW4869 markedly attenuated the pro-osteogenic effect of SCs on PDLSCs. These findings suggest that EVs represent important paracrine mediators of the pro-osteogenic effects of SCs on PDLSCs. In an SC-depleted microenvironment following IAN transection, the osteogenic capacity of PDLSCs was impaired. However, exogenous supplementation with SC-EVs was associated with increased autophagic flux, restored osteogenic differentiation and promoted periodontal tissue regeneration in vivo models. Taken together, these findings suggest that SC-EVs may mediate SC regulation of PDLSC osteogenic differentiation. Among the diverse bioactive cargo of SC-EVs, miRNAs have emerged as important effector molecules because of their conserved roles in post-transcriptional gene regulation. miRNAs are key regulators of periodontal tissue homeostasis, and influence periodontal stem cell differentiation as well as osteoblast and osteoclast function. Osteogenic differentiation of PDLSCs is particularly critical for periodontal tissue repair. miRNAs such as miR-27, miR-29, and miR-199a promote this process [35]. Furthermore, in inflammatory environments, miR-146a can increase PDLSC differentiation by inhibiting NF-κB signaling [36]. In addition, appropriate autophagic activity is required for osteogenic differentiation of PDLSCs [37]. Multiple miRNAs, such as miR-155, miR-128a, and miR-146a, regulate autophagy [38–40]. In this study, small RNA sequencing revealed that miR-210-5p is a highly enriched miRNA in SC-EVs and is a potentially important regulator that coordinates both autophagy and osteogenesis. Mechanistic investigations revealed that miR-210-5p could directly bind to the 3′ UTR of the *DAP1* gene to suppress its expression. DAP1, a member of the DAP family, is closely associated with cell growth and programmed cell death processes such as apoptosis and autophagy. Its activity is highly dependent on the regulatory status of mTOR [26, 41]. During osteogenesis, DAP1 inhibits LC3 lipidation and subsequent autophagosome formation in osteoblasts via the ATG16L1-LC3 axis [42]. Dual-luciferase reporter assays revealed the specific targeting of DAP1 by miR-210-5p. These findings are consistent with a previous report that miR-210-5p regulates autophagy via the PIK3R5/AKT/mTOR pathway [24], and suggest that miR-210-5p may be involved in the regulation of autophagy. Based on these findings, miR-210-5p may represent one functional component of the SC-EV cargo contributing to the observed effects, although other miRNAs and proteins are also likely to be involved.

Autophagy is a critical downstream effector of AMPK signaling in bone metabolism and promotes bone formation through multiple mechanisms [23, 43–46]. For instance, resveratrol prevents post-ovariectomy osteoporosis by activating the AMPK/JNK1 axis, thus restoring osteocyte autophagy and mitigating apoptosis [47]. Similarly, negative pressure wound therapy enhances bone regeneration in critical-sized defects by activating the AMPK-ULK1 pathway to induce autophagy [48]. In addition, activation of PPARβ/δ promotes the osteogenic differentiation of BMMSCs under high-glucose conditions by increasing AMPK phosphorylation, inhibiting mTOR, and enhancing autophagy [23]. Consistent with these observations, our study showed that the SC-depleted microenvironment induced by IAN transection disrupted AMPK–mTOR signaling homeostasis in PDLSCs, suppressed autophagic activity, and consequently impaired osteogenic capacity. In contrast, exogenous SC-EVs, through delivery of miR-210-5p and suppression of DAP1, were associated with increased autophagic activity and partial restoration of the osteogenic differentiation potential of PDLSCs. Moreover, autophagy activation may represent one component of a broader metabolic and cellular response induced by SC-EVs, rather than an isolated causal mechanism. Therefore, although our findings support the involvement of autophagy in the pro-osteogenic effects of SC-EVs, they should not be interpreted as definitive proof that autophagy alone is the sole causal mediator of these effects. Although the AMPK-mTOR-autophagy pathway was prioritized in this study, denervation-associated periodontal dysregulation is likely influenced by multiple signaling and neuroregulatory mechanisms. Therefore, the present findings should be interpreted as supporting the involvement of an AMPK-mTOR-autophagy-related mechanism rather than establishing it as the exclusive pathway.

Limitations of the study.

This study has several limitations. First, the relatively limited small in vivo sample size and the exclusive use of rats, and the acute inferior alveolar nerve transection model may affect the robustness, generalizability, and clinical relevance of the findings, as this model does not fully recapitulate the chronic inflammatory microenvironment of clinical periodontitis. Further validation in large-animal models, chronic disease models, and human-relevant systems is therefore needed. Second, EV characterization in the present study was primarily based on TEM, NTA, canonical EV marker detection, and protein quantification. More rigorous analyses, such as particle-to-protein ratio assessment and advanced EV purity characterization, were not performed, which should be considered when interpreting the specificity and purity of the isolated SC-EV preparations. Third, the SC-EVs used in this study were derived from the RSC96 cell line rather than primary Schwann cells, which remains an important unresolved limitation at present. As immortalized cell lines may differ from primary human Schwann cells in EV cargo composition and biological activity, future studies should validate these findings using primary Schwann cell-derived EVs. Fourth, although our data support the involvement of the SC-EVs/miR-210-5p/DAP1/autophagy-related axis in promoting osteogenesis, this central mechanism needs further confirmation, as the rescue experiments were partial, and autophagy should be interpreted as a functionally relevant associated process rather than a fully established sole causal mechanism. In addition, other EV cargo components and signaling pathways may also contribute to the observed effects, while the long-term safety, stability, biodistribution, immunogenicity, optimal dosage, and administration frequency of SC-EVs remain to be determined. Furthermore, although the present study focused primarily on PDLSCs and osteogenic differentiation, periodontal regeneration is a multifactorial process involving inflammatory regulation, vascularization, immune modulation, cementogenesis, and periodontal ligament organization, and further studies are needed to clarify the effects of SC-EVs on other relevant cells and processes. Notably, SC-EVs may be beneficial beyond direct nerve-injury settings, although this requires validation in non-denervation periodontal defect models. These limitations should be considered when interpreting the translational significance of the present findings.

## Conclusions

In summary, this study revealed that inferior alveolar nerve transection induces progressive Schwann cell depletion and periodontal bone deterioration, accompanied by compromised osteogenic function of periodontal ligament stem cells. Our data support that Schwann cell-derived extracellular vesicles can partially restore osteogenic differentiation, with the miR-210-5p/DAP1/autophagy-related pathway representing one potential mechanism. These findings contribute to the understanding of peripheral nerve-mediated periodontal regeneration and provide a basis for further preclinical investigation of Schwann cell-derived EV-based strategies for periodontal repair.

## Supplementary Information

Supplementary material 1.

## Data Availability

The RNA-seq and small RNA-seq data generated in this study are publicly available in the NCBI Sequence Read Archive (SRA) under BioProject accession numbers PRJNA1374455 and PRJNA1374456.

## References

[1] GBD 2021 Oral Disorders Collaborators. Trends in the global, regional, and national burden of oral conditions from 1990 to 2021: a systematic analysis for the Global Burden of Disease Study 2021. Lancet. 2025;405:897–910. 10.1016/S0140-6736(24)02811-3 40024264

[2] Trindade D, Carvalho R, Machado V, Chambrone L, Mendes JJ, Botelho J. Prevalence of periodontitis in dentate people between 2011 and 2020: A systematic review and meta-analysis of epidemiological studies. J Clin Periodontol. 2023;50:604–26. 10.1111/jcpe.13769 36631982

[3] Nuñez J, Vignoletti F, Caffesse RG, Sanz M. Cellular therapy in periodontal regeneration. Periodontol 2000. 2019, 79: 107–116. 10.1111/prd.12250 30892768

[4] De Lauretis A, Øvrebø Ø, Romandini M, Lyngstadaas SP, Rossi F, Haugen HJ. From Basic Science to Clinical Practice: A Review of Current Periodontal/Mucogingival Regenerative Biomaterials. Adv Sci (Weinh). 2024;11:e2308848. 10.1002/advs.202308848 PMC11077667 38380549

[5] Jessen KR, Mirsky R, Lloyd AC. Schwann Cells: Development and Role in Nerve Repair. Cold Spring Harb Perspect Biol. 2015;7:a020487. 10.1101/cshperspect.a020487 PMC4484967 25957303

[6] Taveggia C. Schwann cells-axon interaction in myelination. Curr Opin Neurobiol. 2016;39:24–9. 10.1016/j.conb.2016.03.006 27089429

[7] Carr MJ, Johnston AP. Schwann cells as drivers of tissue repair and regeneration. Curr Opin Neurobiol. 2017;47:52–7. 10.1016/j.conb.2017.09.003 28963968

[8] Meyers CA, Lee S, Sono T, Xu J, Negri S, Tian Y, et al. A Neurotrophic Mechanism Directs Sensory Nerve Transit in Cranial Bone. Cell Rep. 2020;31:107696. 10.1016/j.celrep.2020.107696 PMC7335423 32460020

[9] Meng L, Yang P, Zhang W, Zhang X, Rong X, Liu H, et al. Brain-derived neurotrophic factor promotes orthodontic tooth movement by alleviating periodontal ligament stem cell senescence. Cell Signal. 2023;108:110724. 10.1016/j.cellsig.2023.110724 37211081

[10] Hao Z, Ren L, Zhang Z, Yang Z, Wu S, Liu G, et al. A multifunctional neuromodulation platform utilizing Schwann cell-derived exosomes orchestrates bone microenvironment via immunomodulation, angiogenesis and osteogenesis. Bioact Mater. 2023;23:206–22. 10.1016/j.bioactmat.2022.10.018 PMC9672134 36439082

[11] Lopez-Verrilli MA, Picou F, Court FA. Schwann cell-derived exosomes enhance axonal regeneration in the peripheral nervous system. Glia. 2013;61:1795–806. 10.1002/glia.22558 24038411

[12] Johnston AP, Yuzwa SA, Carr MJ, Mahmud N, Storer MA, Krause MP, et al. Dedifferentiated Schwann Cell Precursors Secreting Paracrine Factors Are Required for Regeneration of the Mammalian Digit Tip. Cell Stem Cell. 2016;19:433–48. 10.1016/j.stem.2016.06.002 27376984

[13] Parfejevs V, Debbache J, Shakhova O, Schaefer SM, Glausch M, Wegner M, et al. Injury-activated glial cells promote wound healing of the adult skin in mice. Nat Commun. 2018;9:236. 10.1038/s41467-017-01488-2 PMC5770460 29339718

[14] Yamazaki S, Ema H, Karlsson G, Yamaguchi T, Miyoshi H, Shioda S, et al. Nonmyelinating Schwann cells maintain hematopoietic stem cell hibernation in the bone marrow niche. Cell. 2011;147:1146–58. 10.1016/j.cell.2011.09.053 22118468

[15] Zhang X, Jiang X, Jiang S, Cai X, Yu S, Pei G. Schwann cells promote prevascularization and osteogenesis of tissue-engineered bone via bone marrow mesenchymal stem cell-derived endothelial cells. Stem Cell Res Ther. 2021;12:382. 10.1186/s13287-021-02433-3 PMC8261922 34233721

[16] Miceli RT, Chen TY, Nose Y, Tichkule S, Brown B, Fullard JF, et al. Extracellular vesicles, RNA sequencing, and bioinformatic analyses: Challenges, solutions, and recommendations. J Extracell Vesicles. 2024;13:e70005. 10.1002/jev2.70005 PMC11613500 39625409

[17] Wang T, Li W, Zhang Y, Xu X, Qiang L, Miao W, et al. Bioprinted constructs that simulate nerve-bone crosstalk to improve microenvironment for bone repair. Bioact Mater. 2023;27:377–93. 10.1016/j.bioactmat.2023.02.013 PMC10131128 37122897

[18] Wang Y, Qu F, Wu Y, Lan K, Shen Y, Wu Z, et al. Peripheral nerves modulate the peri-implant osteogenesis under type 2 diabetes through exosomes derived from schwann cells via miR-15b-5p/Txnip signaling axis. J Nanobiotechnol. 2025;23:51. 10.1186/s12951-025-03160-0 PMC11773925 39875954

[19] Wu Z, Pu P, Su Z, Zhang X, Nie L, Chang Y. Schwann Cell-derived exosomes promote bone regeneration and repair by enhancing the biological activity of porous Ti6Al4V scaffolds. Biochem Biophys Res Commun. 2020;531:559–65. 10.1016/j.bbrc.2020.07.094 32811642

[20] Mori MA, Ludwig RG, Garcia-Martin R, Brandão BB, Kahn CR. Extracellular miRNAs: From Biomarkers to Mediators of Physiology and Disease. Cell Metab. 2019;30:656–73. 10.1016/j.cmet.2019.07.011 PMC6774861 31447320

[21] Spreca A, Rambotti MG, Rende M, Saccardi C, Aisa MC, Giambanco I, et al. Immunocytochemical localization of S-100b protein in degenerating and regenerating rat sciatic nerves. J Histochem Cytochem. 1989;37:441–6. 10.1177/37.4.2926122 2926122

[22] Jones RE, Salhotra A, Robertson KS, Ransom RC, Foster DS, Shah HN, et al. Skeletal Stem Cell-Schwann Cell Circuitry in Mandibular Repair. Cell Rep. 2019;28:2757–e27665. 10.1016/j.celrep.2019.08.021 PMC6820052 31509739

[23] Chen M, Jing D, Ye R, Yi J, Zhao Z. PPARβ/δ accelerates bone regeneration in diabetic mellitus by enhancing AMPK/mTOR pathway-mediated autophagy. Stem Cell Res Ther. 2021;12:566. 10.1186/s13287-021-02628-8 PMC8567548 34736532

[24] Liu W, Jiang D, Gong F, Huang Y, Luo Y, Rong Y, et al. miR-210-5p promotes epithelial-mesenchymal transition by inhibiting PIK3R5 thereby activating oncogenic autophagy in osteosarcoma cells. Cell Death Dis. 2020;11:93. 10.1038/s41419-020-2270-1 PMC7002725 32024814

[25] Lv XW, He ZF, Zhu PP, Qin QY, Han YX, Xu TT. miR-451-3p alleviates myocardial ischemia/reperfusion injury by inhibiting MAP1LC3B-mediated autophagy. Inflamm Res. 2021;70:1089–100. 10.1007/s00011-021-01508-4 34633468

[26] Koren I, Reem E, Kimchi A. Autophagy gets a brake: DAP1, a novel mTOR substrate, is activated to suppress the autophagic process. Autophagy. 2010;6:1179–80. 10.4161/auto.6.8.13338 PMC3039719 20818178

[27] Nanci A, Bosshardt DD. Structure of periodontal tissues in health and disease. Periodontol 2000. 2006, 40: 11–28. 10.1111/j.1600-0757.2005.00141.x 16398683

[28] Salhotra A, Shah HN, Levi B, Longaker MT. Mechanisms of bone development and repair. Nat Rev Mol Cell Biol. 2020;21:696–711. 10.1038/s41580-020-00279-w PMC7699981 32901139

[29] Itoyama T, Yoshida S, Tomokiyo A, Hasegawa D, Hamano S, Sugii H, et al. Possible function of GDNF and Schwann cells in wound healing of periodontal tissue. J Periodontal Res. 2020;55:830–9. 10.1111/jre.12774 32562261

[30] Cabrera-Pastor A. Extracellular Vesicles as Mediators of Neuroinflammation in Intercellular and Inter-Organ Crosstalk. Int J Mol Sci. 2024;25:7041. 10.3390/ijms25137041 PMC11241119 39000150

[31] Xia W, Xie J, Cai Z, Liu X, Wen J, Cui ZK, et al. Damaged brain accelerates bone healing by releasing small extracellular vesicles that target osteoprogenitors. Nat Commun. 2021;12:6043. 10.1038/s41467-021-26302-y PMC8519911 34654817

[32] Isaac R, Reis FCG, Ying W, Olefsky JM. Exosomes as mediators of intercellular crosstalk in metabolism. Cell Metab. 2021;33:1744–62. 10.1016/j.cmet.2021.08.006 PMC8428804 34496230

[33] Li Z, Liang Y, Pan K, Li H, Yu M, Guo W, et al. Schwann cells secrete extracellular vesicles to promote and maintain the proliferation and multipotency of hDPCs. Cell Prolif. 2017;50:e12353. 10.1111/cpr.12353 PMC6529079 28714175

[34] Su Y, Gao Q, Deng R, Zeng L, Guo J, Ye B, et al. Aptamer engineering exosomes loaded on biomimetic periosteum to promote angiogenesis and bone regeneration by targeting injured nerves via JNK3 MAPK pathway. Mater Today Bio. 2022;16:100434. 10.1016/j.mtbio.2022.100434 PMC9519612 36186848

[35] Luan X, Zhou X, Trombetta-eSilva J, Francis M, Gaharwar AK, Atsawasuwan P, et al. MicroRNAs and Periodontal Homeostasis. J Dent Res. 2017;96:491–500. 10.1177/0022034516685711 PMC5453493 28068481

[36] Hung PS, Chen FC, Kuang SH, Kao SY, Lin SC, Chang KW. miR-146a induces differentiation of periodontal ligament cells. J Dent Res. 2010;89:252–7. 10.1177/0022034509357411 20110513

[37] Yin Y, Tian BM, Li X, Yu YC, Deng DK, Sun LJ, et al. Gold nanoparticles targeting the autophagy-lysosome system to combat the inflammation-compromised osteogenic potential of periodontal ligament stem cells: From mechanism to therapy. Biomaterials. 2022;288:121743. 10.1016/j.biomaterials.2022.121743 36030103

[38] D’Adamo S, Alvarez-Garcia O, Muramatsu Y, Flamigni F, Lotz MK. MicroRNA-155 suppresses autophagy in chondrocytes by modulating expression of autophagy proteins. Osteoarthritis Cartilage. 2016;24:1082–91. 10.1016/j.joca.2016.01.005 PMC4875787 26805019

[39] Lian WS, Ko JY, Wu RW, Sun YC, Chen YS, Wu SL, et al. MicroRNA-128a represses chondrocyte autophagy and exacerbates knee osteoarthritis by disrupting Atg12. Cell Death Dis. 2018;9:919. 10.1038/s41419-018-0994-y PMC6134128 30206206

[40] Chen G, Gao X, Wang J, Yang C, Wang Y, Liu Y, et al. Hypoxia-induced microRNA-146a represses Bcl-2 through Traf6/IRAK1 but not Smad4 to promote chondrocyte autophagy. Biol Chem. 2017;398:499–507. 10.1515/hsz-2016-0211 27845876

[41] Yahiro K, Tsutsuki H, Ogura K, Nagasawa S, Moss J, Noda M. DAP1, a negative regulator of autophagy, controls SubAB-mediated apoptosis and autophagy. Infect Immun. 2014;82:4899–908. 10.1128/IAI.02213-14 PMC4249318 25183729

[42] Gao M, Du Z, Dong Q, Su S, Tian L. DAP1 regulates osteoblast autophagy via the ATG16L1-LC3 axis in Graves’ disease-induced osteoporosis. J Orthop Surg Res. 2023;18:711. 10.1186/s13018-023-04171-z PMC10512661 37735431

[43] Steinberg GR, Hardie DG. New insights into activation and function of the AMPK. Nat Rev Mol Cell Biol. 2023;24:255–72. 10.1038/s41580-022-00547-x 36316383

[44] Alers S, Löffler AS, Wesselborg S, Stork B. Role of AMPK-mTOR-Ulk1/2 in the regulation of autophagy: cross talk, shortcuts, and feedbacks. Mol Cell Biol. 2012;32:2–11. 10.1128/MCB.06159-11 PMC3255710 22025673

[45] Wang JF, Mei ZG, Fu Y, Yang SB, Zhang SZ, Huang WF, et al. Puerarin protects rat brain against ischemia/reperfusion injury by suppressing autophagy via the AMPK-mTOR-ULK1 signaling pathway. Neural Regen Res. 2018;13:989–98. 10.4103/1673-5374.233441 PMC6022469 29926825

[46] Zhang S, Zha H, Chen Q, Li N, Zheng C, Xie T, et al. DISC1 Protects Against Zika Virus Infection and Long-Term Neurological Damage Through AMPK-mTOR-Mediated Autophagy. Nat Commun. 2025;16:9841. 10.1038/s41467-025-64809-w PMC12595038 41203603

[47] Wei L, Chai S, Yue C, Zhang H, Li J, Qin N. Resveratrol protects osteocytes against oxidative stress in ovariectomized rats through AMPK/JNK1-dependent pathway leading to promotion of autophagy and inhibition of apoptosis. Cell Death Discov. 2023;9:16. 10.1038/s41420-023-01331-2 PMC9867734 36681672

[48] Zhang S, Xie Y, Yan F, Zhang Y, Yang Z, Chen Z, et al. Negative pressure wound therapy improves bone regeneration by promoting osteogenic differentiation via the AMPK-ULK1-autophagy axis. Autophagy. 2022;18:2229–45. 10.1080/15548627.2021.2016231 PMC9466622 34964701

