# Supplementary material for: Schwann cell-derived extracellular vesicles promote periodontal tissue regeneration
Source: J Nanobiotechnology. 2026 Jul 26;24:973. doi: 10.1186/s12951-026-04848-7 (PMC13685203; doi:10.1186/s12951-026-04848-7)
Supplement: Supplementary file 1 [file 12951_2026_4848_MOESM1_ESM.docx]

**Table S1. RT-PCR primer sequences**

| **Gene** | **Forward Primer sequences (5'-3')** | **Reverse Primer sequences (5'-3')** |
| --- | --- | --- |
| Runx2 | CAGACACAATCCTCCCCACC | GCCAGAGGCAGAAGTCAGAG |
| ALP | GGCTGGAGATGGACAAGTTC | ACGCCACACAAGTAGGCAGT |
| Col I | TCTGACTGGAAGAGCGGAGAG | GAGTGGGGAACACACAGGTCT |
| BSP | ACAACGGAGAAGAAGCCGAG | TCGTGGTGCCATAACTGGTC |
| OPN | TCCAGGAGTTTCCCTGTTTC | TGACCTTGATAGCCTCATCG |
| BMP2 | TGGGTTTGTGGTGGAAGTGGC | TGGATGTCCTTTACCGTCGTG |
| LC3B | TCCCCGGTCACTCACTC | AGGTGGCTGTGTTGGCT |
| SQSTM1/P62 | GACTTGGTCGCCTTCTCC | ATGCTTCGTGCCTCCTG |
| Beclin1 | ATCCTGGACCGTGTCACCATCCAGG | GTTGAGCTGAGTGTCCAGCTGG |
| AMPK | TTGCGTGTGCGAAGGAAGAACC | CCGATCTCTGTGGAGTAGCAGTCC |
| mTOR | TTGGAGAACCAGCCCATAAGA | ATGAGATGTCGCTTGCTTGATAA |
| U6 | CCTGCTTCGGCAGCACA | AACGCTTCACGAATTTGCGT |
| DAP1 | CAAGCGCGCAGCATCAGTC | GTGGACAACTACGCCACGAC |
| GAPDH | TGATGGGTGTGAACCACGAG | CCCTTCCACGATGCCAAAGT |
| β-actin | TGCTATGTTGCCCTAGACTTCG | GTTGGCATAGAGGTCTTTACGG |
| rno-miR-451-3p | GTCGTATCCAGTGCAGGGTCCGAGGTATTCGCACTGGATACGACAGCAGC |  |
| rno-miR-296-5p | GTCGTATCCAGTGCAGGGTCCGAGGTATTCGCACTGGATACGACACAGGA |  |
| rno-miR-210-6p | GTCGTATCCAGTGCAGGGTCCGAGGTATTCGCACTGGATACGAC CAGTGT |  |
| rno-miR-451-3p | GCCCATGGTAATGGTTCTCTT | AGTGCAGGGTCCGAGGTATT |
| rno-miR-210-5p | ACAGCCACTGCCCACAGC | AGTGCAGGGTCCGAGGTATT |

**Supplemental Figures and Figure Legends**

**Figure S1. Morphological characterization and identification of PDLSCs.** (A) Morphology of PDLSCs, and staining images of Oil Red O. Scale bars: 100 μm. (B) Surface marker expression of PDLSCs from the sham-operated group. (C) Surface marker expression of PDLSCs from the denervated group. (D) Effect of denervation on Von Kossa staining of PDLSCs. Scale bars: 200 μm.

**Figure S2. Schwann cell depletion correlates with periodontal bone loss after denervation.** (A) Immunohistochemical staining for S100 in the periodontal ligament on postoperative day 1. (B) Immunohistochemical staining for S100 in the periodontal ligament on postoperative day 5. (C) Immunohistochemical staining for S100 in the periodontal ligament on postoperative day 7. (D) Immunohistochemical staining for S100 in the periodontal ligament on postoperative day 14. n = 6 per group. Statistical analysis: Panel A, B and D unpaired t-test; panel C, Mann–Whitney U test. Data are presented as mean ± SD.

**Figure S3. Effect of denervation on the expression of genes related to the AMPK-mTOR-autophagy pathway in PDLSCs.** (A) Differentially expressed gene analysis of transcriptomes from PDLSCs of denervated and sham-operated groups. (B) Effect of denervation on the expression of AMPK-mTOR pathway-related genes in PDLSCs. (C) Effect of denervation on the expression of autophagy-related genes in PDLSCs. Statistical analysis: panel B, Mann-Whitney test and unpaired t-test; panel C, Mann–Whitney U test for Beclin1, unpaired t-test for LC3 and Welch’s t-test for SQSTM1. Data are presented as mean ± SD.

**Figure S3. Effect of SC-EVs on PDLSCs.** (A) Immunofluorescence characterization of RSC96 cells. Red: p75NTR; Green: S100; Blue: DAPI. Scale bars: 20 μm. (B) Effect of SC-EVs on the expression of AMPK-mTOR pathway-related genes in PDLSCs. (C) Effect of SC-EVs on the expression of autophagy-related genes in PDLSCs. (D) miR-210-5p promotes the expression of autophagy-related genes in PDLSCs. Statistical analysis: panel B, unpaired t-test; panel C, Welch’s t-test for SQSTM1 and unpaired t-test for others; panel D, unpaired t-test. Data are presented as mean ± SD.
